# Hydrogen‐Deuterium Exchange Defines Ligand‐Induced Conformational Changes to the Class III Biotin Protein Ligase from *Saccharomyces cerevisiae*

**DOI:** 10.1002/cbic.202500439

**Published:** 2025-09-17

**Authors:** Louise M. Sternicki, Tara L. Pukala, Kamila J. Pacholarz, Perdita Barran, Grant W. Booker, Steven W. Polyak, Kate L. Wegener

**Affiliations:** ^1^ School of Biological Sciences The University of Adelaide Adelaide South Australia 5005 Australia; ^2^ School of Physics Chemistry and Earth Sciences The University of Adelaide Adelaide South Australia 5005 Australia; ^3^ Manchester Institute of Biotechnology The University of Manchester Manchester M1 7DN UK; ^4^ Institute for Photonics and Advanced Sensing (IPAS) The University of Adelaide Adelaide South Australia 5005 Australia; ^5^ Present address: Institute for Biomedicine and Glycomics Grifith University Gold Coast Queensland 4222 Australia

**Keywords:** biotin protein ligase, hydrogen‐deuterium exchange, mass spectrometry, native mass spectrometry, structural biology

## Abstract

Biotin protein ligase (BPL) catalyzes the covalent attachment of biotin onto biotin‐dependent enzymes, where it functions as an essential cofactor. Eukaryotic BPLs are distinct due to the presence of a large N‐terminal extension to the conserved catalytic domain and C‐terminal cap. No high‐resolution structures of a eukaryotic BPL have been solved; however, previous functional studies revealed the N‐terminal extension interacts with the biotinylation substrate. Mass spectrometry (MS) and complementary techniques were utilized to investigate the structure of the yeast *Saccharomyces cerevisiae* BPL (*Sc*BPL). Lower resolution techniques suggested holo‐*Sc*BPL had a more compact structure and sampled fewer conformational states. In addition, solution‐phase and a charge state dependent gas‐phase stabilization was observed. Hydrogen‐deuterium exchange (HDX) MS provided experimental validation of the AlphaFold predicted structure of *Sc*BPL, with a folded domain structurally homologous to a glutamine amidotransferase identified in the N‐terminal extension, and a mostly homologous catalytic domain to that of other species’ BPLs. Further HDX analyses identified localized conformational changes in the *Sc*BPL active site and N‐terminal domain that occur concomitantly with ligand binding. These data provide novel insights into the unique structure of a class III BPL and how ligands influence this structure for catalysis of protein biotinylation.

## Graphical abstract

Hydrogen‐deuterium exchange mass spectrometry validated AlphaFold structural prediction of the class III biotin protein ligase (BPL) from Saccharomyces cerevisiae, and revealed a structured domain in the N‐terminal extension characteristic to this class of BPL. Ligand binding to *Sc*BPL induced localized conformational changes in the active site and N‐terminal domain, with reduced conformational sampling and increased stability.

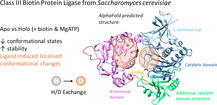

## 1 Introduction

**Figure 1:**
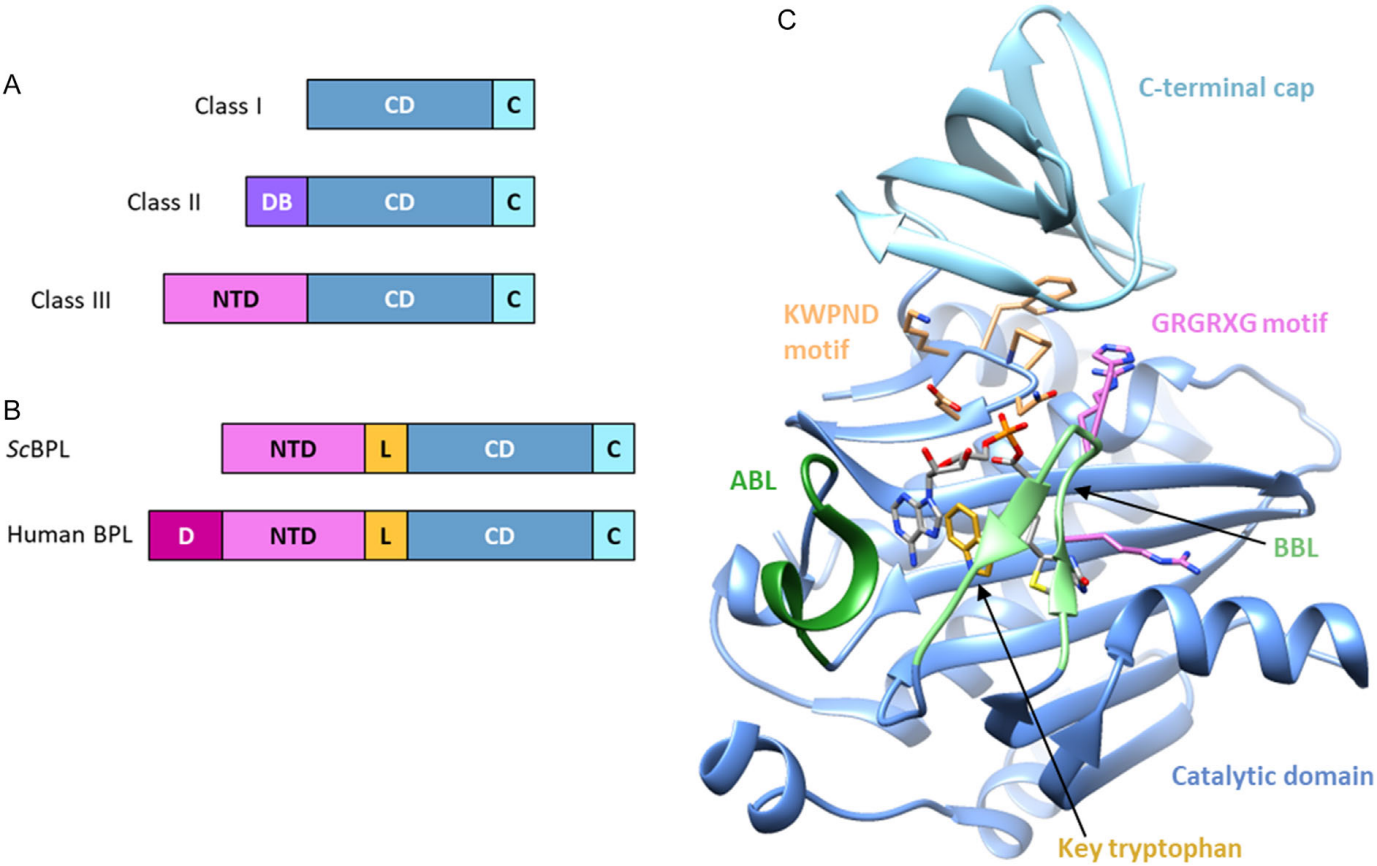
A) The domain architecture of the three structural classes of BPLs. Class I enzymes (e.g., *M. tuberculosis* ^[^ ^8^ ^]^) contain the minimal structural requirements for biotinylation, consisting of the catalytic domain (CD, dark blue) and C‐terminal cap (C, light blue). In addition to the minimal functional structure, Class II enzymes (e.g., *S. aureus, E. coli* ^[^ ^10^ ^]^) contain a N‐terminal DNA binding domain (DB, purple) that enables transcriptional regulation of biotin supply, whilst the class III enzymes (e.g., *S. cerevisiae*, humans) contain a larger N‐terminal domain extension (NTD, pink) that assists with substrate recognition. B) Examples of the domain architecture for two class III BPLs. The *S. cerevisiae* BPL (*Sc*BPL) is hypothesized to contain a short protease susceptible linker (L, orange) between the N‐terminal domain and catalytic domain. The human BPL also contains an additional 160‐residue disordered N‐terminal region (D, dark pink). C) Example structure of a class I BPL bound to the reaction intermediate biotinyl‐5′‐AMP from *M. tuberculosis* (PDB: 4OP0)^[^ ^8^ ^]^ showing key structural features. Figure adapted from ref. [3].

Biotin (vitamin B7, vitamin H) is essential for all forms of life as it is an indispensable cofactor for key metabolic enzymes, where it acts as a transient carrier of carboxyl groups during metabolic reactions.^[^ ^1^ ^]^ Biotin protein ligase (BPL) is the enzyme responsible for the post‐translational attachment of biotin onto biotin‐dependent enzymes.^[^ ^2^ ^,^ ^3^ ^]^ BPL carries out protein biotinylation via a two‐step mechanism. The first step involves the binding of substrates biotin and MgATP in adjacent pockets where they ligate to form the reaction intermediate biotinyl‐5′‐AMP.^[^ ^4^ ^]^ This holo–enzyme complex then forms a heterologous protein–protein interaction with a substrate biotin‐dependent enzyme such that the biotinyl moiety can be transferred from the reaction intermediate onto a target lysine sidechain that has been precisely inserted into the BPL active site.^[^ ^5^ ^]^ This core reaction mechanism is conserved throughout the biological world as BPLs can often recognize and biotinylate protein substrates from various other species (reviewed^[^ ^6^ ^]^). Structural biology has shown conformational changes within BPLs are also an important feature of the reaction mechanism. For example, some BPLs, such as from *Mycobacterium tuberculosis, Staphylococcus aureus, Escherichia coli*, and humans, require biotin to bind prior to ATP, as the binding of biotin induces a structural rearrangement in the active site to form the ATP binding site.^[^ ^7^, ^9^ ^–^ ^11^ ^]^ This includes the ordering of a previously disordered loop (termed the biotin‐binding loop), which re‐positions a key tryptophan residue to accommodate π–π stacking interactions with the purine rings of ATP (**Figure** **1**).^[^ ^10^ ^,^ ^12^ ^]^ The ordering of additional loops in the active site upon ligand binding further stabilizes the binding of ligands and protects the labile reaction intermediate from hydrolysis and dissociation.^[^ ^10^ ^,^ ^13^ ^]^

BPLs across all kingdoms of life can be divided into three structural classes (Figure 1). The class I and II enzymes are the simplest enzymes and are mainly found in archaea, bacteria, and plants. Class I enzymes contain a conserved catalytic domain and C‐terminal cap, which together constitute the minimal structural requirements for protein biotinylation.^[^ ^4^ ^,^ ^8^ ^,^ ^14^ ^,^ ^15^ ^]^ In addition to these modules, class II BPLs also contain an N‐terminal DNA binding domain,^[^ ^10^ ^,^ ^12^ ^,^ ^16^ ^]^ which allows transcriptional regulation of biotin‐related genes, such as those involved in biotin transport and synthesis in response to intracellular and extracellular biotin concentrations (reviewed^[^ ^17^ ^]^). In contrast, the class III enzymes from fungi, mammals and insects contain a much larger N‐terminal extension to the catalytic domain and C‐terminal cap. This extension has no homology with the class II N‐terminal domain and no predicted DNA binding capability. While the class I and II enzymes have been thoroughly characterized, with multiple structures solved from each class, the class III enzymes have been less well studied. To date, no high‐resolution structure of a class III enzyme has been experimentally determined, and no definitive function has been assigned to the large N‐terminal extension.

Previous biochemical studies on class III BPLs have concentrated on the yeast (*Saccharomyces cerevisiae*) and human enzymes. Limited proteolysis experiments and functional assays of truncation mutants predicted the presence of a structured domain in the N‐terminal extension of both these enzymes that was essential for activity.^[^ ^11^ ^,^ ^18^ ^–^ ^20^ ^]^ For yeast BPL, this domain is located between residues 1–240,^[^ ^18^ ^]^ whereas in the human homolog, it lies between residues 160 and 300 (Figure 1).^[^ ^11^ ^,^ ^19^ ^]^ This N‐terminal domain has been hypothesized to promote biotinylation by specifically recognizing the protein substrate, as evidenced by yeast‐two hybrid^[^ ^20^ ^]^ and surface plasmon resonance^[^ ^11^ ^]^ experiments on human BPL. In addition, nuclear magnetic resonance (NMR) spectroscopy binding studies suggested that residues 1–160 of human BPL may also interact with the protein substrate,^[^ ^21^ ^]^ although this region is predicted to be disordered. The specificity of the enzyme–substrate interaction is proposed to provide a “substrate verification” activity, whereby only appropriate biotin‐dependent enzymes are selected for biotinylation.^[^ ^3^ ^]^ Several studies have demonstrated that class III BPLs have the highest affinity toward the biotin‐dependent enzymes they natively biotinylate (reviewed^[^ ^3^ ^]^) in preference to protein substrates originating from different species^[^ ^19^ ^,^ ^22^, ^23^ ^–^ ^24^ ^]^ or other biotin‐dependent enzymes within a species.^[^ ^25^ ^]^ Alternate isoforms of human BPL, the result of alternate translation initiation at different N‐terminal residues (M1 versus M57), have also been reported to have varying affinities for the five different mammalian biotin‐dependent enzymes.^[^ ^26^ ^]^ This provided further evidence of a role for the N‐terminal extension in substrate recognition and selectivity. Despite this, no specific structural features or regions in the conserved protein substrates have been identified as conferring selective recognition by class III BPLs. Only one X‐ray crystal structure of a BPL in complex with its protein substrate has been solved.^[^ ^5^ ^]^ However, this was a class I BPL from the thermophilic bacteria *Pyrococcus horikoshii* and, hence, does not contain an N‐terminal extension. While the interaction of the catalytic domain with the protein substrate is predicted to be conserved among different BPL classes, the structure and positioning of the class III N‐terminal domain in this interaction remains unknown.

Aside from the active site conformational structuring of loops described above, additional structural changes in the N‐terminal extension are proposed to accompany ligand binding in class III BPLs. This has been hypotheszed based on limited proteolysis experiments of the yeast BPL*,* for which a reduction in proteolysis was observed upon the addition of the ligands biotin and MgATP.^[^ ^18^ ^]^ These low‐resolution data implied ligand‐induced conformational changes alter the structure of the protease sensitive region (residues 240–260) such that it is less accessible to proteases.^[^ ^18^ ^]^ This region is proposed to form a solvent accessible linker that lies between the hypothesized N‐terminal domain and the catalytic domain (Figure 1). Together, these available data imply that the BPL alters from an open, apo state that is receptive to binding ligands to a more compact holo‐enzyme in complex with biotinyl‐5′‐AMP that is primed for interaction with an appropriate protein substrate. Further structural and functional investigation of ligand‐induced rearrangements in *Sc*BPL are required to map the precise locations of these and other potential conformational changes, how these regions change structurally, and to understand how these contribute to the specificity of the biotinylation reaction of class III BPLs.

Uncovering the mechanism of class III BPLs will be crucial for further understanding the human condition known as multiple carboxylase deficiency (MCD). This severe metabolic disease arises due to inherited defects in the human BPL (also known as holocarboxylase synthetase) that results in reduced biotinylation of all five human biotin‐dependent enzymes. Missense mutations often decrease the affinity for biotin (*K*_M_ mutations) (reviewed^[^ ^6^ ^]^). Hence, MCD patients often respond favorably to oral supplements of biotin. Of particular relevance are certain MCD missense mutations that do not alter the *K*_M_ for biotin^[^ ^27^, ^28^ ^–^ ^29^ ^]^ and, consequently, do not respond to supplemental biotin therapy.^[^ ^30^, ^31^ ^–^ ^32^ ^]^ Many of the relevant mutations for these biotin‐unresponsive MCD variants map to residues 160–300 in the unique N‐terminal extension of the human BPL. Surface plasmon resonance studies have demonstrated these mutations reduce the affinity for the protein substrate by increasing the dissociation rate, thereby inhibiting the enduring interaction of the protein–protein complex necessary for biotinyl‐transfer.^[^ ^11^ ^]^ Determining the structure and function of this N‐terminal extension in class III BPLs will advance our understanding of these biotin‐unresponsive MCD mutants and provide potential avenues for treatment.

In this report, we characterize the ligand‐induced structural changes in the class III BPL from the prototypical eukaryote *S. cerevisiae* (*Sc*BPL) using hydrogen‐deuterium exchange (HDX). During HDX, hydrogen atoms in the peptide backbone that are surface exposed, located in dynamic protein regions or not involved in the structural hydrogen‐bonding network, can undergo exchange to the heavier deuterium isotope.^[^ ^33^, ^34^ ^–^ ^35^ ^]^ Liquid chromatography‐mass spectrometry (LC‐MS) analysis of the digested deuterated peptides provides the amount and sequence location of the incorporated deuterium. Mapping this HDX data onto a model of *Sc*BPL generated by AlphaFold provided new insights into the conformational changes that accompany ligand binding. HDX was performed alongside orthogonal structural techniques, such as circular dichroism (CD) and ion mobility‐mass spectrometry (IM‐MS), to further investigate protein structural changes in response to ligand binding. Finally, we discuss how these findings extend our current knowledge of biotin metabolism and its significance in understanding defects in BPL activity in human disease.

## 2 Results

### 2.1 Apo‐ and Holo‐ScBPL had Similar Gross Structures

An investigation into the conformational changes that accompany ligand binding was performed using a variety of biochemical and biophysical approaches. Non‐liganded (i.e., apo‐*Sc*BPL) used in this study was prepared and characterized as previously described (Figure S1 and S2, Supporting Information). Holo‐*Sc*BPL was subsequently obtained by incubating apo enzyme with biotin and MgATP (Figure S1 and S2, Supporting Information). Ligand treatment caused the native MS *Sc*BPL ion peaks to shift to higher *m/z* values with a corresponding measured mass of 77,817 Da (expected mass 77,817 Da), consistent with the binding of biotinyl‐5′‐AMP to monomeric *Sc*BPL (Figure S2 and Table S1, Supporting Information).

**Figure 2:**
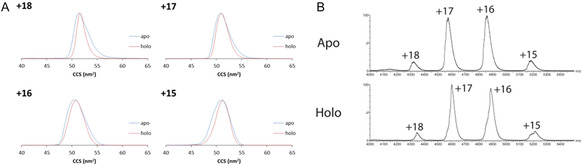
A) ^TW^CCS_N2_ distributions of apo‐ (blue) and holo‐*Sc*BPL (red) across the four charge states observed by IM‐MS, and B) the corresponding nMS spectrum. The standard error of the IM‐MS technique is 5%.^[^ ^37^ ^]^

CD and native IM‐MS were initially employed to compare secondary structure and overall globular structure differences in *Sc*BPL once ligands bind. The CD spectra of apo‐ and holo‐*Sc*BPL were essentially superimposable, suggesting no substantial secondary structural changes between the two states (Figure S3, Supporting Information). Both apo‐ and holo‐*Sc*BPL contained a maximum near 193 nm and minima at 208 and 222 nm indicative of α‐helical secondary structure, as well as signal around 218 nm consistent with the presence of some β‐sheet structure. Native MS revealed apo‐ and holo‐*Sc*BPL were structurally similar as the charge state distributions (+15 to +18) and the predominant charge states (+16 and +17) were the same for both enzymes (**Figure** **2**). IM‐MS also confirmed apo‐ and holo‐*Sc*BPL had similar overall structures. IM‐MS separates molecules based on their mobility through buffer gas (termed drift time), which is influenced by size, shape, and mass. The mobility measurement can be converted into a collision cross section (CCS) that represents the rotationally averaged surface area of the molecule and, hence, its size and shape.^[^ ^36^ ^]^ The average ^TW^CCS_N2_ of apo‐ and holo‐*Sc*BPL across all charge states were found to be essentially the same (^TW^CCS_N2_ apo‐*Sc*BPL: 50.72 nm^2^, ^TW^CCS_N2_ holo‐*Sc*BPL: 50.82 nm^2^) (**Table** **1**), with any variation within the standard error of 5% for the technique.^[^ ^37^ ^]^ This indicated that there was no difference in the average overall shape or structure of apo‐ and ligand‐bound *Sc*BPL. The ^TW^CCS_N2_ distribution curves for holo‐*Sc*BPL were slightly narrower compared to apo‐*Sc*BPL and consistently had smaller widths at half height across all charge states (Figure 2, Table S2, Supporting Information). While ^TW^CCS_N2_ distributions can be sensitive to the nMS peak widths, there does not appear to be notable differences in the nMS peak widths between apo‐ and holo‐*Sc*BPL, with IM drift times extracted over similar *m/z* ranges for both *Sc*BPL states. Therefore, the ^TW^CCS_N2_ distributions suggested apo‐*Sc*BPL may sample a greater variety of spatial conformations; however, the level of variation is close to the limit of resolution of the MS instrument and IM‐MS technique. Despite this, CD and IM‐MS did not indicate any large‐scale structural rearrangements (i.e., overall changes in shape or domain reorganizations) upon ligand binding to *Sc*BPL.

**Table 1:** Collision cross sections (CCS) reveal no difference in the overall structures of apo‐ and holo‐*Sc*BPL.

	^TW^CCS_N2_ [nm^2^]				Average ^TW^CCS_N2_ [nm^2^]
	15+	16+	17+	18+	
Apo	50.85	49.99	50.49	51.54	50.72
Holo	50.85	50.39	50.49	51.54	50.82

### 2.2 Ligand‐Binding Increased ScBPL Stability

**Figure 3:**
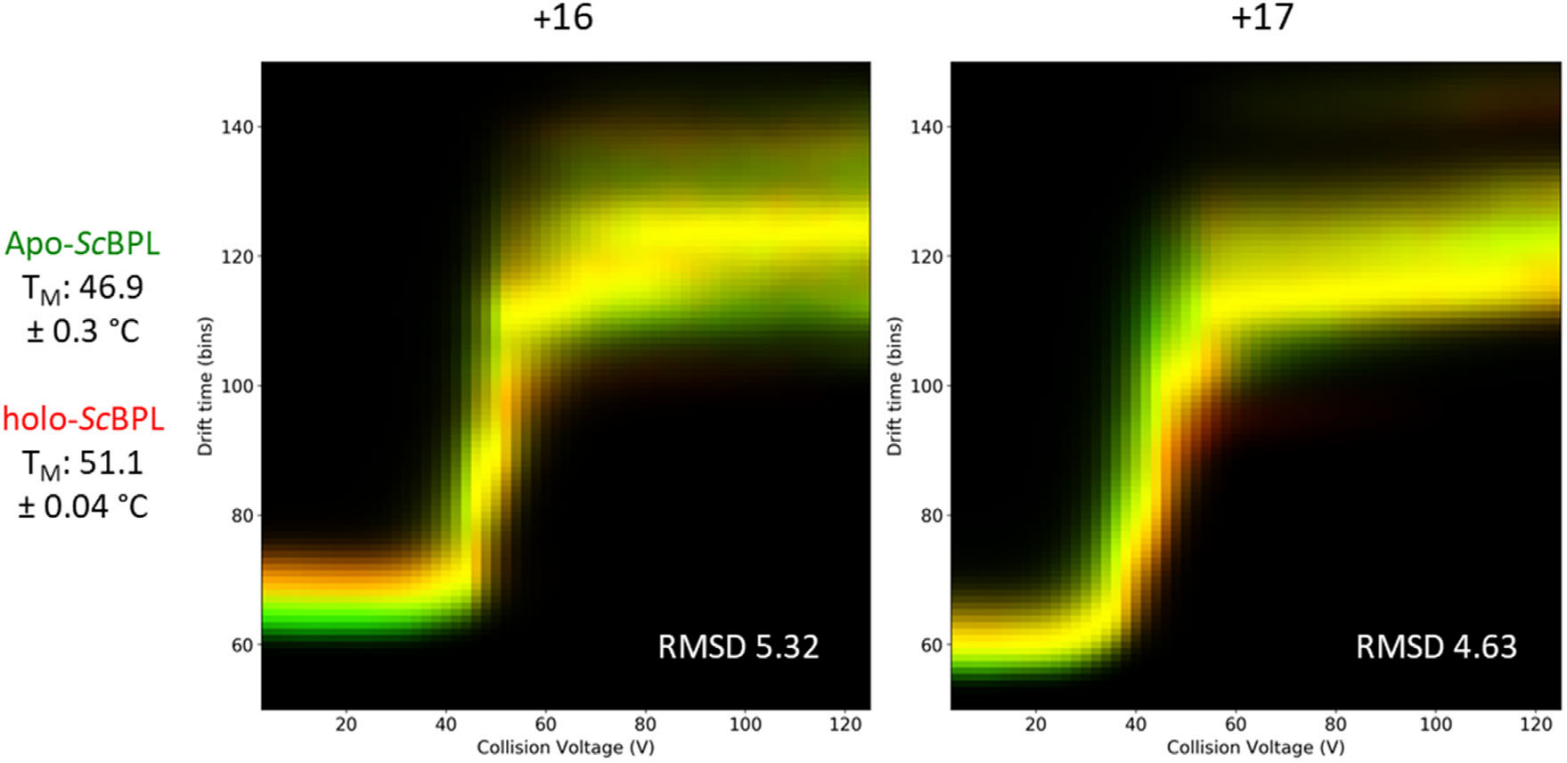
Comparisons of the stability of apo‐ and holo‐*Sc*BPL by in solution thermal denaturation assays (*T*_M_ annotated on left of figure) and collision‐induced unfolding MS (CIU‐MS). During CIU‐MS, increasing voltages (*x*‐axis) are applied to unfold the protein, causing an increase in protein size and, therefore, drift time (*y*‐axis). Shown is an overlay of the CIU data for the two most intense charge states, +17 and + 16, where green indicates the drift time of apo‐*Sc*BPL and red the drift time of holo‐*Sc*BPL as collisional voltage is increased. Yellow coloring shows the overlap in drift time, and therefore similar unfolding, between apo‐ and holo‐*Sc*BPL.

The stabilities of apo‐*Sc*BPL and holo‐*Sc*BPL were compared via both gas‐phase collision induced unfolding‐MS (CIU‐MS) and in‐solution thermal denaturation assays. CIU‐MS involves sequentially increasing the trap collision energy to promote protein unfolding whilst utilizing drift time as a measurement of protein denaturation. This technique allows the determination of the different transitions a protein adopts as it unfolds, whilst measuring its overall stability in the gas phase. Both of these can inform on protein stability.^[^ ^38^ ^]^ Here, CIU‐MS revealed ligand binding stabilized *Sc*BPL for one of the two charge states analyzed (**Figure** **3** and S4, Supporting Information). Overlays of the unfolding curves for the +17 charge state of apo‐ and holo‐*Sc*BPL revealed the holo‐*Sc*BPL unfolding curve is slightly right‐shifted toward higher voltages (Figure 3). The magnitude of this change was comparable to other published examples of ligands stabilizing proteins.^[^ ^39^ ^,^ ^40^ ^]^ However, CIU‐MS unfolding curves for the +16 charge state aligned (Figure 3). Therefore, CIU‐MS revealed a charge state dependent stabilization of *Sc*BPL upon ligand binding, with no stabilization evident for the +16 charge state and a small increase in gas‐phase stabilization for the +17 charge state. Ligand binding did not alter the unfolding pathway, as both apo‐ and holo‐*Sc*BPL passed through two short‐lived transition states before reaching similar extended conformations at voltages of 50–60 V (Figure 3 and S4, Supporting Information). This further demonstrated a high degree of structural homology between apo‐ and holo‐*Sc*BPL. Given the disparate results regarding charge state dependent gas‐phase stabilization with ligand binding, solution state thermal denaturation assays were also employed to assess *Sc*BPL stabilization with ligand binding. Solution thermal denaturation assays revealed holo‐*Sc*BPL had a higher melting temperature (*T*_M_) of 51.1 °C ± 0.04 °C compared with apo‐*Sc*BPL with a *T*_M_ of 46.9 °C ± 0.3 °C (*p* = 0.006, Figure 3), and that *Sc*BPL unfolded via a single transition despite the prediction of two separate structural domains (Figure S5, Supporting Information). Therefore, while CIU‐MS of the 16+ charge state indicated no stabilization, CIU‐MS of the 17+ charge state and thermal denaturation assays supported an increased stability of *Sc*BPL following ligand binding.

### 2.3 Analysis of the ScBPL AlphaFold Model

**Figure 4:**
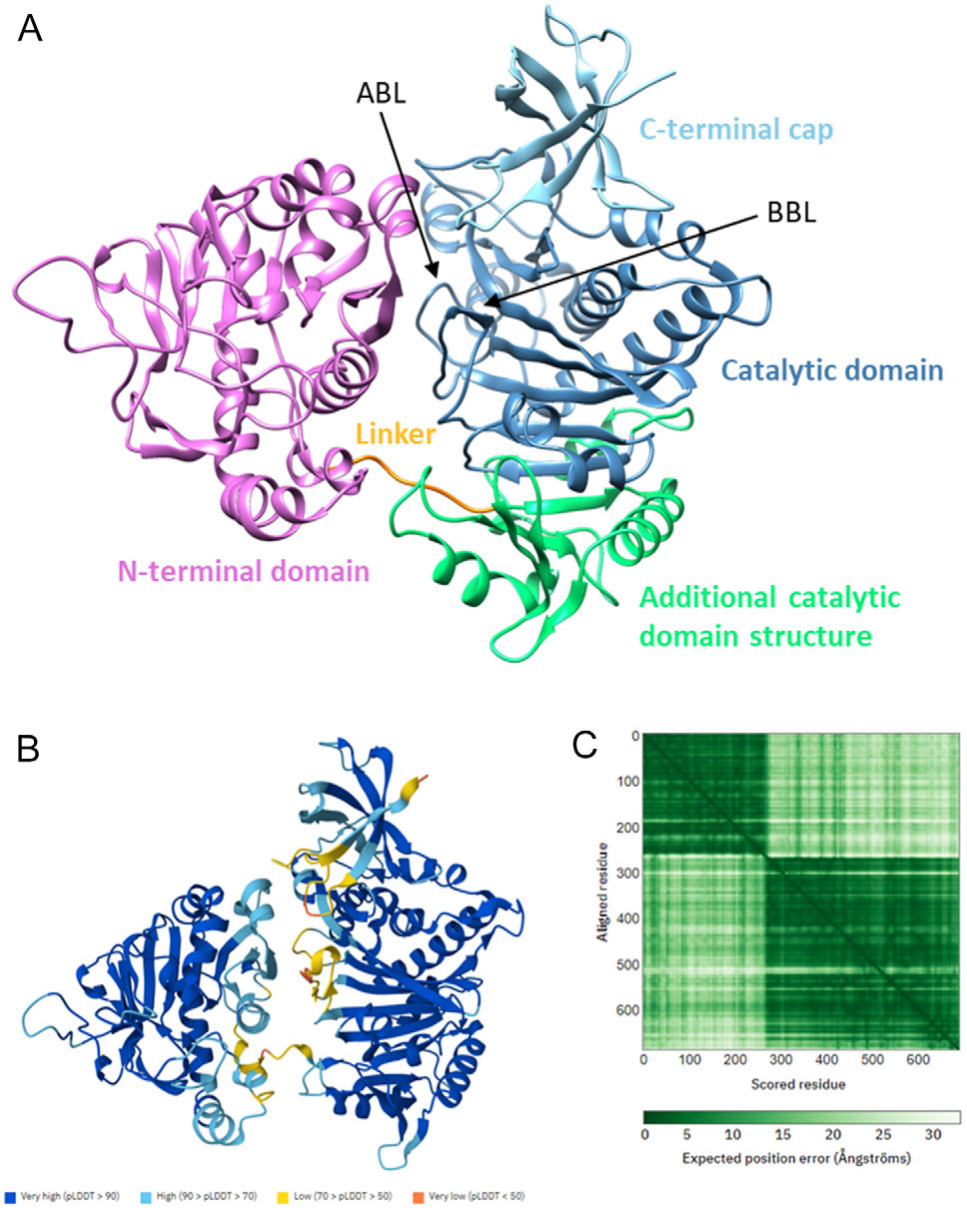
A) Structure of *Sc*BPL as proposed by AlphaFold (AF‐P48445‐F1‐v4, UniProt P48445),^[^ ^41^ ^,^ ^42^ ^]^ and confidence analysis of this structure by way of B) the per‐residue model confidence score (pLDDT) and C) the predicted aligned error for assessing inter‐domain accuracy. BBL = biotin binding loop; ABL = adenylate binding loop.

In the absence of a high‐resolution *Sc*BPL structure, the AlphaFold structural prediction model of *Sc*BPL was analyzed (**Figure** **4**).^[^ ^41^ ^,^ ^42^ ^]^ The majority of the structure was modeled with high confidence (>90%), while some of the more flexible loops (including those in the BPL active site that are often not resolved in other species’ crystal structures, i.e., the biotin binding and adenylate binding loops), the potential inter‐domain linking and interacting regions, and some parts of the C‐terminal cap had lower confidence (50–70%). Analysis of this structural prediction against previously empirically derived structures of other species BPLs (comprising class I and II enzymes) revealed a homologous, well overlaid BPL catalytic domain and C‐terminal cap (Figure 4A, blue) as expected, since all known BPLs possess similar catalytic sites necessary to catalyze the universally conserved protein biotinylation mechanism (Figure S6, Table S3, Supporting Information).^[^ ^6^ ^,^ ^43^ ^]^ Furthermore, sequence motifs and catalytic residues that are important for BPL function, including the GRGRXG (located on the biotin‐binding loop) and KWPND (located at the back of the substrate binding pocket) motifs, both essential for biotin binding, and the key tryptophan residue (W430 in *Sc*BPL, located on the biotin‐binding loop) required for *π*‐*π* stacking interactions with ATP, were conserved and correctly positioned within the model when compared to empirical BPL structures from other species.^[^ ^44^, ^45^ ^–^ ^46^ ^]^ Interestingly, another small, structured region (Figure 4A, green) elongated the catalytic domain in *Sc*BPL compared with other BPL counterparts, similar to the N‐terminal DNA binding domain extension of class II BPLs. However, this folded region of *Sc*BPL did not resemble the structure of the class II N‐terminal DNA binding domains and was, therefore, not predicted to bind DNA, particularly as eukaryotic organisms with class III BPLs do not synthesize biotin and, hence, don’t require feedback regulation of biotin synthesis. This structure has not previously been predicted by homology modeling, and the function or necessity of this region for activity has not been assessed.

A structured domain was predicted between residues 1 to ≈260 of the N‐terminal extension of *Sc*BPL. The location of this domain was compatible with previous limited proteolysis experiments that predicted a folded domain in the first 240 residues of *Sc*BPL,^[^ ^18^ ^]^ with protease susceptibility between residues 240 and 260. Analysis of this structure revealed that this region is the final surface exposed alpha helix in this domain before the linking region connecting it to the larger catalytic domain. Homology modeling predicted this folded N‐terminal domain shared structural homology (23% amino acid sequence identity) to a glutamine amidotransferase (GATase) fold (for comparison the amino acid sequence identity for the well‐conserved C‐terminal catalytic domain with *E. coli* BPL was 22%).^[^ ^3^ ^,^ ^47^ ^]^ Unexpectedly, the catalytic triad of residues (cysteine, histidine, and glutamic acid) responsible for GATase enzymatic function was conserved and correctly positioned in the *Sc*BPL N‐terminal domain (residues C89, H215, E217) (Figure S7A, Supporting Information). Within GATases, these residues catalyze the conversion of glutamine to glutamate and ammonia such that the ammonia can be utilized in other enzymatic subunits of the GATase protein complex for further catalytic reactions.^[^ ^48^ ^,^ ^49^ ^]^ Hence, the relevance of this residue triad in BPL is unknown. *Sc*BPL was incapable of producing glutamate in vitro when incubated with glutamine (and MgCl_2_ to aid GATase catalysis), as determined by ^1^H 1D NMR spectroscopy (Figure S7B, Supporting Information). Similarly, in vitro *Sc*BPL biotinylation activity was unaltered in the presence of glutamine (Figure S8, Supporting Information). Thus, *Sc*BPL appears to not contain GATase activity in vitro despite having a domain homologous to a GATase.

The *Sc*BPL AlphaFold structure provided the first insight into the location and positioning of the N‐terminal and C‐terminal catalytic domains of a class III BPL relative to each other. There was a short linking region (residues 257–276) such that the N‐terminal domain sits adjacent to the substrate binding active site of the catalytic domain (Figure 4). Analysis of the *Sc*BPL AlphaFold predicted aligned error does reveal that there is lower confidence in the prediction of the relative locations of the two domains, suggesting that the domains may have some degree of mobility relative to each other. Empirical validation is required to validate the positioning of the domains in this structural model.

### 2.4 HDX‐MS Revealed Ligand‐Induced Conformational Changes to ScBPL

HDX‐MS was subsequently utilized to further investigate the structure of *Sc*BPL in both the apo‐ and holo‐states. Following LC‐MS of the pepsin digested HDX reactions, 149 unique peptides were detected in all replicates of the unlabeled control reactions of both apo‐ and holo‐*Sc*BPL, providing 85.5% sequence coverage (Figure S9, Supporting Information).

**Figure 5:**
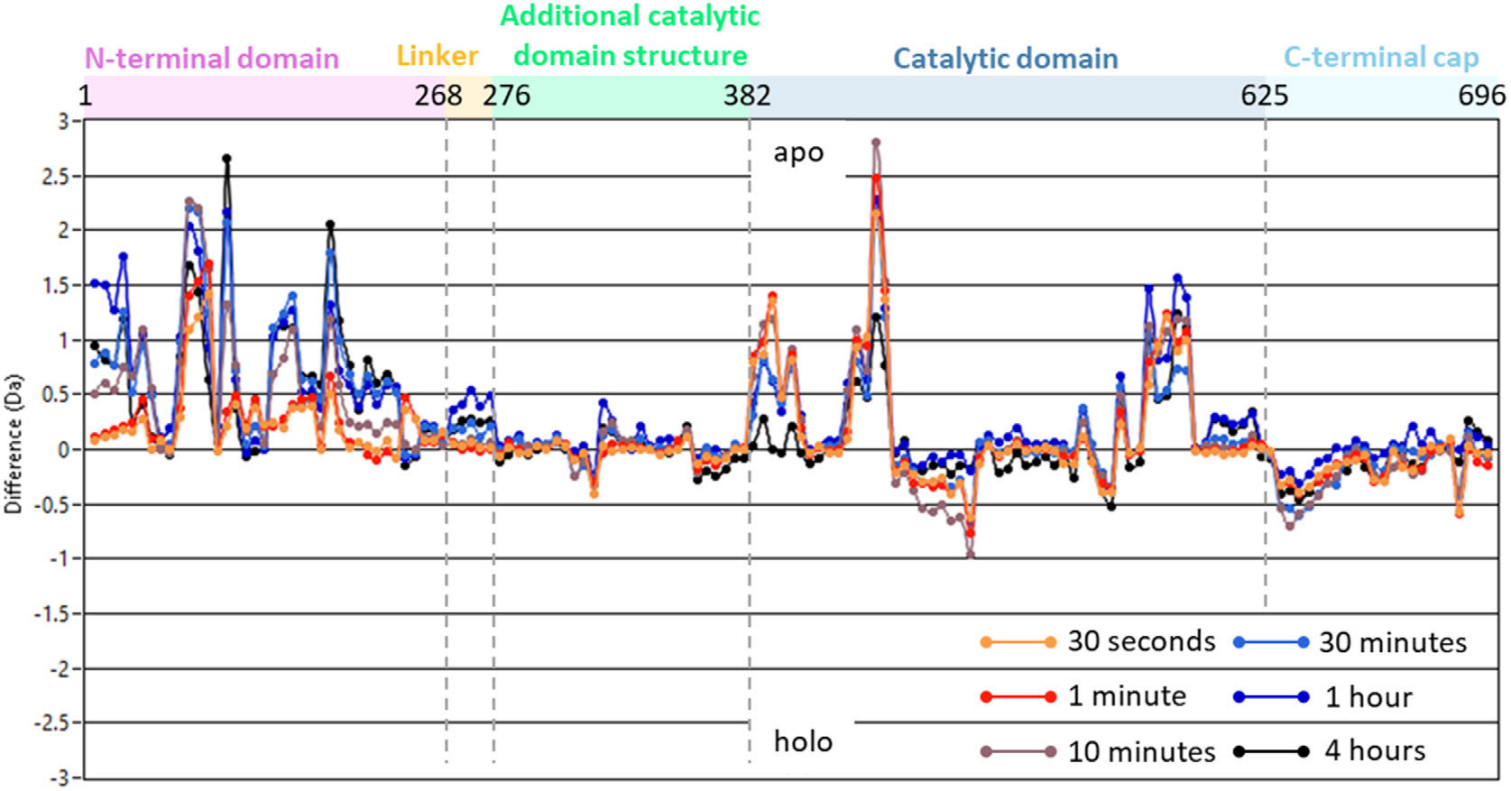
Apo‐*Sc*BPL incorporated more deuterium across majority of the *Sc*BPL sequence than holo‐*Sc*BPL. The difference in the uptake of deuterium between apo‐ and holo‐*Sc*BPL for each peptide identified after 30 s (orange), 1 min (red), 10 min (gray), 30 min (light blue), 1 h (dark blue), and 4 h (black) exposure to deuterium. *Sc*BPL domains, and the corresponding residue number, are indicated at the top. Where peptides crossed the boundary of two domains, they were included in the structural domain that the majority of the peptide was located in.

Deuterium incorporation within apo‐*Sc*BPL was limited to specific regions, with the remaining protein having little to no deuterium uptake (Figure S10, Supporting Information). This suggested the protein is primarily folded with a few surface‐exposed or dynamic regions. The N‐terminal extension (residues 1–220) contained multiple stretches of sequence greater than 15 residues in length that did not incorporate deuterium, implying these are buried within a folded structure, supporting the presence of a structured domain within this extension. The 20 residues previously identified in the literature as being susceptible to proteases (240–260) were not observed in the LC‐MS coverage, and therefore, confirmation of the surface accessibility of this region could not be determined. Comparison of the deuterium uptake across the *Sc*BPL sequence between apo‐ and holo‐*Sc*BPL revealed apo‐*Sc*BPL incorporated more deuterium than holo‐*Sc*BPL across most of the sequence (**Figure** **5** and S11, Supporting Information). This suggested that structural changes occur upon ligand binding that reduce the ability of holo‐*Sc*BPL to incorporate deuterium. This is consistent with the IM‐MS data that suggested apo‐*Sc*BPL has a more open structure that samples more conformational states and that the enzyme rigidifies upon ligand binding. There were fewer regions that incorporated more deuterium in the holo state compared to the apo state, including residues 313–328, 443–460, 525–528, and 624–640, all located in the catalytic domain and C‐terminal cap (Figure S11, Supporting Information). This suggested there are few *Sc*BPL regions that become more surface exposed or are liberated from structural interaction networks by the binding of the ligands, such that these regions are available to exchange with deuterium.

**Figure 6:**
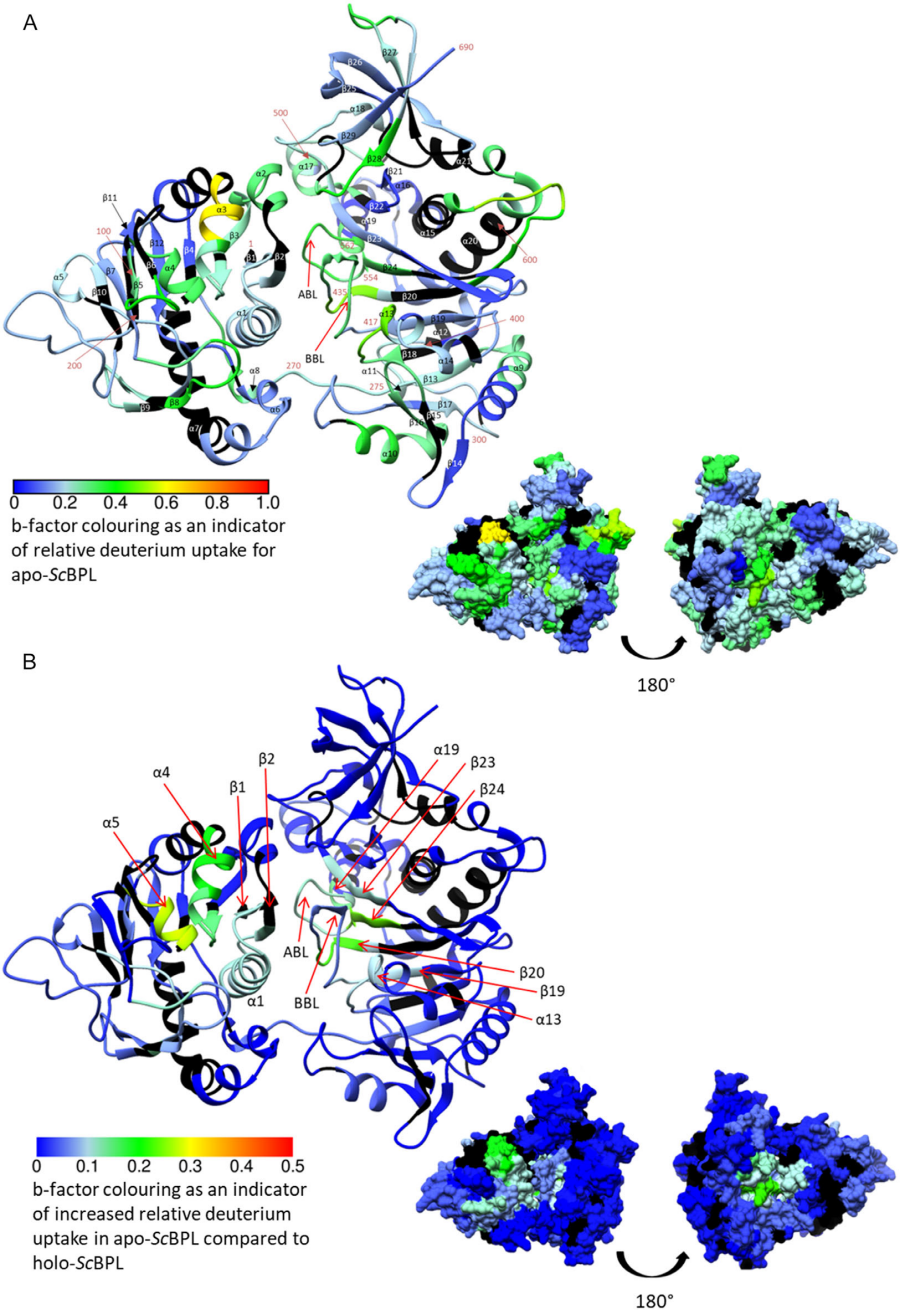
A) Deuterium incorporation for apo‐*Sc*BPL and B) regions of *Sc*BPL that have reduced deuterium uptake upon ligand (biotin and MgATP) binding. All relative deuterium incorporation from 1 h of deuterium exposure was converted to b‐factors for each residue (within DynamX analysis software), and the b‐factor results are mapped onto both ribbon and surface fill representations of the *Sc*BPL AlphaFold structure (AF‐P48445‐F1‐v4, UniProt P48445).^[^ ^41^ ^,^ ^42^ ^]^ Rainbow color schemes indicate either A) the level of deuterium uptake in apo‐*Sc*BPL (dark blue indicates no deuterium exchange through to light blue, green and yellow representing increasing levels of deuterium incorporation; colored according to deuterium incorporation b‐factor with the following key: 0 blue, 0.2 light blue, 0.4 green, 0.6 yellow, 0.8 orange and 1 red), or B) the difference in deuterium incorporation between apo‐ and holo‐*Sc*BPL (dark blue indicates no difference, while light blue and green indicate increasing amounts of deuterium incorporation in apo‐*Sc*BPL compared with holo‐*Sc*BPL; colored according to deuterium incorporation b‐factor with the following key: 0 blue, 0.1 light blue, 0.2 green, 0.3 yellow, 0.4 orange, 0.5 red). Black indicates regions not included in the HDX LC‐MS coverage. Alpha helix and beta strands are labeled as structurally assigned in Chimera.^[^ ^63^ ^]^ BBL = biotin binding loop; ABL = adenylate binding loop.

The apo‐*Sc*BPL HDX results were mapped onto the AlphaFold predicted structure of *Sc*BPL to structurally view the relative deuterium uptake (indicated by b‐factor) and allow experimental validations of the proposed structural model (**Figure** **6**A). This species had the greater deuterium incorporation rates (compared with the ligand bound holo‐state), suggesting that it was the most open and conformationally variable state of *Sc*BPL. It should be noted that the resolution of the technique allowed the location of deuterium incorporation to be localized to a short peptide (up to 25 residues in length), rather than individual residues. Regions of the catalytic domain with greater deuterium incorporation were logically located on predicted exposed surfaces, loop regions and in the active site, including regions that are seemingly adjacent to the N‐terminal domain and proposed to be involved in interdomain interactions, supporting potential mobility of the two domains relative to each other (Figure 6A). There was little to no deuterium incorporation observed in the inner catalytic domain core. Specifically, deuterium incorporation occurred in the active site ligand binding pockets, including within β‐strands β20 and β24 that form the back surface of the active site pockets along with the biotin‐binding loop (residues 421–435), part of the adenylate‐binding loop (residues 554–562), and another disordered loop that helps form the biotin‐binding pocket (residues 388–394). These data are consistent with other BPLs where analogous loops are disordered in crystal structures of the apo‐enzyme but become ordered upon ligand binding.^[^ ^8^ ^,^ ^10^ ^,^ ^12^ ^]^ Other surface loops of *Sc*BPL (residues 443–460 that includes part of α15 and β20 that the loop connects to; residues 495–528 including α17, α18, and part of β23 that lies within a core β‐sheet; and residues 604–611 including the adjacent ends of α20 & α21 that this linker joins) also incorporated deuterium, compatible with the dynamic nature of these unstructured loops. The majority of deuterium incorporation within the catalytic domain was located on the side of the protein containing the active site. There was also evidence of deuterium incorporation within the C‐terminal cap domain (comprised of surface exposed β‐strands) and the elongated catalytic domain relative to the BPLs from other classes, specifically the linker from the N‐terminal domain into β13, β13 itself and the start of α9, and the loop connecting residue 313 to β16, along with β16 itself and α10.

Some areas in the C‐terminal catalytic domain were not included in the LC‐MS coverage and, therefore, the HDX uptake in these regions is unknown (Figure 6A). This predominantly included sections of β18, β20, and β24 that form the part of the β‐sheet protein core, and parts of α12, α15, α20, and α21 that are located on the opposite side of the catalytic domain to the ligand‐binding site. There were also areas that appeared surface exposed within the model that did not incorporate deuterium, such as residues 566–578 immediately following the adenylate‐binding loop that form the exposed α19 and linker to α20 of the domain's core, and part of the extended catalytic domain relative to BPLs from other classes (residues 291–306 including part of α9, β14, and the interlinking regions). The absence of deuterium exchange in these seemingly surface accessible regions suggested that they had lower solvent accessibility than predicted from the AlphaFold model. This discrepancy could arise from inaccuracies in AlphaFold's modeling of surface‐exposed or flexible regions,^[^ ^50^ ^]^ or from slower conformational dynamics in this region preventing exchange. These possibilities highlight the importance of integrating experimental data with predictive models to accurately interpret protein structure and dynamics. Additional experiments will be necessary to understand this further.

Deuterium uptake was also mapped onto the N‐terminal domain (Figure 6A). Interestingly, whilst many regions of this small domain incorporated deuterium, they only underwent very low levels of exchange, consistent with a folded domain structure. The region of highest deuterium uptake across the entire BPL's structure (including the catalytic domain) was residues 62–68 located toward the N‐terminal end of α3. The other regions of high deuterium uptake, above the low levels observed across most of this domain, included residues 39–61 (α2, β2–3, α3), residues 90–109 (α4, β5), residues 129–153 (surface loop into β8–9), and residues 261–276 (linker connecting the N‐terminal domain into the catalytic domain). These regions were located on one side of the protein structure (similar to the catalytic domain) and clustered around the one large surface exposed region that sits adject to the catalytic domain active site in the AlphaFold model. The accuracy of the boundaries of some of these regions of higher deuterium incorporation are limited due to the bounding regions not being covered in the LC‐MS coverage. Additionally, other regions in the N‐terminal domain were not covered by LC‐MS analysis (Figure 6A), including part or all of β1, β2, β4, β6, β10, β11, α3, and α7. Several regions did not incorporate deuterium, including the core β‐sheet structure, N‐terminal residues 1–34, and residues 220–234 that were located adjacent to the catalytic domain and may be involved in inter‐domain interactions. Additionally, residues 176–198 were seemingly located on a surface exposed loop; however, they underwent no to minor levels of deuterium exchange, suggesting that these residues may be less dynamic and/or involved in interaction networks. Overall, the HDX supported the proposed structure for *Sc*BPL, revealing that the protein is mostly folded with a structured domain in the N‐terminal extension that is linked to an elongated catalytic domain relative to class I and II BPLs.

### 2.5 HDX and Homology Modeling Revealed Locations of Ligand‐Induced Conformational Changes

The difference in deuterium incorporation between apo‐ and holo‐*Sc*BPL was mapped onto the predicted *Sc*BPL structure (by way of b‐factor as an indicator of relative deuterium uptake) to identify regions of *Sc*BPL that underwent structural alteration upon ligand binding (Figure 6B). Overall, there was a general reduction in deuterium incorporation by *Sc*BPL following ligand addition (Figure 5, S11 and S12, Supporting Information). This is consistent with the enzyme becoming more rigid, thereby reducing surface exposure, increasing bonding networks, and preventing flexible regions from undergoing deuterium exchange. As expected, regions surrounding the ligand binding sites had reduced deuterium uptake following ligand binding (Figure 6B). These regions included the loop that helps form the biotin‐binding pocket located between β18 and α13 (residues 387–395), along with the biotin‐binding loop (residues 417–435) (Figure S12, Supporting Information). Residues 554–562 from the adenylate‐binding loop, as well as parts of the β‐sheet that form the base of the ligand‐binding pocket (particularly β19, β20, β23, and β24) and connect the various substrate binding site loops, also had reduced deuterium uptake in holo‐*S*cBPL.

Regions of the N‐terminal domain with reduced deuterium uptake due to ligand binding were mostly located on a single surface patch on the side of the protein domain that sits adjacent to the substrate binding site of the catalytic domain (Figure 6B). This was the same part of the domain that had some of the greatest levels of deuterium exchange in the apo‐*Sc*BPL structure (Figure 6B). The regions of the largest difference in deuterium incorporation between apo‐ and holo‐*Sc*BPL were residues 54–68, located on β3, α3, and the interconnecting loop, and residues 90–98 from α4 and the following linker (Figure 6B and S12, Supporting Information). Additionally, the N‐terminal residues 6–35, comprising β1, α1, and β2 had differences in deuterium uptake, albeit to a smaller extent, between the apo and holo states. In contrast, some of the less structured loops and linkers within the N‐terminal domain and the linker connecting this domain to the catalytic domain had only low level differences in deuterium uptake between apo‐ and holo‐*Sc*BPL. These HDX data together with structural modeling of *Sc*BPL provided details on areas of localized conformational changes that occurred with ligand binding.

## 3 Discussion

The absence of an empirically determined high resolution structure of a class III BPL has hindered structural insights into the unique N‐terminal extension and its role in biotinylation. Here, we utilized structural mass spectrometry and complementary techniques to validate the predicted AlphaFold structure of the class III enzyme *Sc*BPL and also investigated conformational changes that occur with the binding of the ligands biotin and MgATP. CD spectroscopy suggested the core secondary structure elements remained the same, while HDX (and to some extent IM‐MS, within the limits of resolution) revealed that holo‐*Sc*BPL had reduced flexibility and/or mobility consistent with the stabilization of ligand binding loops and core structures, rather than large scale rearrangements. Additionally, there was an increased stability of holo‐*Sc*BPL as measured by solution thermal denaturation assays and CIU‐MS of the +17 charge state of *Sc*BPL, however, gas phase stabilization of *Sc*BPL was charge state dependent. Mapping the smaller ligand‐induced structural rearrangements identified by HDX‐MS onto the *Sc*BPL structural model revealed they were situated around the active site of the catalytic domain and on a surface patch of the N‐terminal domain that is located adjacent to and facing the catalytic domain active site (discussed further below). These conformational changes are predicted to be subtle localized structural rearrangements such as loop or helix rigidification or a reduction in protein dynamics, rather than a reorganization of the whole enzyme, explaining why the lower‐resolution techniques that assess the conformationally averaged overall structure (IM‐MS and CD) only suggested potential small changes.

The *Sc*BPL AlphaFold structure revealed, and deuterium uptake data supported, a slightly extended but otherwise homologous catalytic domain and a homologous C‐terminal cap to that of other species BPLs, with this well conserved structural fold between different BPLs necessary to maintain biotinylation activity.^[^ ^6^ ^,^ ^43^ ^]^ The N‐terminal extension had sequence homology with and, therefore, a predicted analogous structure to a glutamine amidotransferase (GATase). These findings were consistent with previous limited proteolysis studies that initially mapped a structured N‐terminal domain in the first 240 residues.^[^ ^18^ ^]^ GATase‐like structures and function have not previously been linked to biotinylation, with no known requirement for ammonium or glutamate (the products generated by GATases) for BPL activity. Despite the models predicting the N‐terminal domain maintains correctly positioned GATase catalytic residues, *Sc*BPL appeared to have no functional GATase activity in vitro, potentially as GATases are generally only active in the presence of the relevant partner synthase substrates.^[^ ^51^, ^52^, ^53^ ^–^ ^54^ ^]^ Conservation of all three catalytic residues was maintained amongst seven example fungal and two additional animal class III BPL sequences aligned (Figure S13, Supporting Information), while at least two of these catalytic residues (H215 and E217 in *Sc*BPL) were conserved in the remaining four sequences compared. The N‐terminal domain's affinity for glutamine (the substrate of GATases) requires investigation and will help elucidate whether the substrate specificity of this domain was altered with domain recruitment. Furthermore, mutation of the GATase catalytic residues conserved in *Sc*BPL could delineate the importance of these residues for biotinylation and/or maintaining the structural GATase‐like fold of this domain.

This AlphaFold *Sc*BPL model also provided the first structural insights into the relative alignment of the N‐ and C‐terminal domains, whereby the N‐terminal domain sits adjacent to the active site of the C‐terminal catalytic domain, presumably enabling the N‐terminal domain to interact with biotin domain substrates as proposed in previous biochemical studies.^[^ ^11^ ^,^ ^18^ ^–^ ^20^ ^]^ HDX revealed that some of the interdomain regions were shielded from deuterium incorporation, as expected for protein–protein interacting regions, while others, such as those around the active site, still incorporated deuterium. This may suggest structural flexibility and mobility of the domains relative to each other, as suggested by AlphaFold's higher predicted aligned error and lower confidence in the relative positioning of the two domains, or inaccuracies in predicting relative domain localization which is a documented weakness for AlphaFold.^[^ ^55^ ^]^ While the AlphaFold models aid contextualization of the HDX results, caution must be exercised to avoid reliance on the absolute accuracy of the models. The HDX analysis provides empirical evidence of seemingly surface exposed regions that do not undergo deuterium exchange and, therefore, may be alternate potential sites of interdomain interactions. The empirical determination of a high resolution structure of a class III BPL, particularly that from *S. cerevisiae,* is required for further confirmation of these structural insights proposed.

HDX was utilized to investigate the subtle structural changes to *Sc*BPL concurrent with ligand binding that result in an increase in stability and a reduction in proteolysis (previously reported^[^ ^18^ ^]^). First, the reduction of HDX in the catalytic domain active site can be explained by 1) these residues undergoing bonding interactions with the ligands and/or reaction intermediate and 2) the binding of ligands sterically occludes these residues from solvent. Second, the surface loops in the active site that have reduced HDX following ligand binding (due to structural rearrangement and/or interactions with the ligands) are analogous to the biotin‐binding loop (residues 418–434 in *Sc*BPL, residues 116–124 in *Ec*BPL) and the adenylate‐binding loop (residues 555–578 in *Sc*BPL, residues 212–234 in *Ec*BPL) of other BPLs, with structural studies of other species’ BPLs revealing these loops undergo disordered‐to‐ordered transitions to form the necessary binding pockets to allow the sequential binding of biotin followed by ATP.^[^ ^10^ ^,^ ^12^ ^,^ ^13^ ^]^ Hence, the HDX provides evidence for similar ordered‐binding mechanisms to be maintained in *Sc*BPL (this has not previously been confirmed). The stabilization of additional loops around the active site and their involvement in ligand interactions can also explain the reduced deuterium uptake in the third loop identified that also forms part of the biotin binding pocket (residues 386–395).

Small but measurable structural rearrangements induced by ligand binding were also identified in the N‐terminal domain. These allosteric changes that reduced deuterium incorporation were centered on a small surface patch located adjacent to the substrate binding site on the C‐terminal domain. The function of this surface is unknown; however, it is postulated that it may be implicated in priming the N‐terminal extension to interact with the biotin domain to form an enduring complex for protein biotinylation to occur, with the interdomain interacting regions not previously determined.^[^ ^11^ ^,^ ^20^ ^,^ ^21^ ^]^ Conservation of the sequence of the highest difference in deuterium uptake (residues 62–68) was poor across eukaryotic BPL sequences outside of fungal sequences (Figure S13, Supporting Information), while the other N‐terminal regions with reduced deuterium uptake in the holo state were conserved to variable extents between the class III BPL sequences compared. Mutagenesis of this N‐terminal domain surface, HDX in the presence of ligands and a biotin domain substrate, or a high‐resolution structure of a class III BPL‐biotin domain complex, would shed light on the function of this surface and the relevance of the ligand‐induced rearrangements in the N‐terminal domain for biotinylation.

The *Sc*BPL models and structural information produced here can be utilized to understand the mechanism behind N‐terminal mutations that cause MCD but are not *K*_M_ mutations and, hence, do not respond to supplemental biotin treatment. Two common N‐terminal domain mutations are L216R and L237P,^[^ ^27^ ^,^ ^56^ ^,^ ^57^ ^]^ which correspond to residues A52 and S79 respectively in the N‐terminal domain of *Sc*BPL (according to sequence alignments^[^ ^58^ ^]^ and AlphaFold model structural overlays). A52 is located in the middle of the core‐located β3 strand of the N‐terminal domain, while S79 is located at the C‐terminal end of α3, on the surface exposed side with its side chain directed outwards (Figure S14, Supporting Information). Mutation L216R (*Sc*BPL A52) would introduce a positively charged side‐chain into the core of the N‐terminal domain, while replacement of S79 with proline would disrupt proper formation of helix α3, changes that are expected to be highly disruptive for domain folding. Helix α3 was seemingly surface located; however, the C‐terminal portion that was observed by LC‐MS coverage did not incorporate deuterium, suggesting it may be significantly involved in interactions or structural networks. Hence, these mutations are likely to disrupt the N‐terminal domain structure (and possibly the overall BPL structure), potentially rationalizing the reduced affinity for the biotin domain substrates previously observed,^[^ ^11^ ^,^ ^20^ ^,^ ^21^ ^]^ contributing to the pathogenic loss of BPL activity.

Here, *Sc*BPL was employed as a model protein to investigate the structure of class III BPLs and their ligand‐induced conformational changes that coincide with biotin and MgATP substrate binding. Integration of structural MS techniques with the AlphaFold predicted structure of *Sc*BPL revealed novel structural insights into *Sc*BPL, including confirming the presence of a structured domain in the N‐terminal extension with conserved homology but not in vitro enzymatic activity of a GATase, and an elongated C‐terminal catalytic domain relative to the catalytic domains of class I and II BPLs. While ligand binding did not appear to result in large‐scale gross structural changes, low resolution techniques and HDX‐MS suggested that holo‐*Sc*BPL had a more compact structure and sampled fewer conformational states. Additionally, holo‐*Sc*BPL had increased solution stability, despite the gas phase stabilization with ligand binding appearing charge state dependent. Localized conformational changes that occurred within the catalytic domain active site and N‐terminal domain upon ligand binding were also identified. We are now working to further validate this structural information using empirically derived data from higher‐resolution structural techniques such as X‐ray crystallography or cryo‐electron microscopy to provide the first experimental structure of a eukaryotic class III BPL.

## 4 Experimental Section

4.1

4.1.1

##### Recombinant Protein Production of Apo‐ and Holo‐ScBPL

Unliganded (i.e., apo) *Sc*BPL was produced as previously reported.^[^ ^24^ ^,^ ^59^ ^]^ Successful purification of apo‐*Sc*BPL was confirmed utilizing both a biotinyl‐transferase assay that employs a previously reported streptavidin‐blot method (Figure S1, Supporting Information)^[^ ^59^ ^,^ ^60^ ^]^ as well as native nESI MS as outlined below (Figure S2, Supporting Information).

##### Native Nano‐Electrospray Ionization Ion Mobility‐Mass Spectrometry (Native nESI IM‐MS)

Native nESI IM‐MS was completed^[^ ^24^ ^]^ with holo‐*Sc*BPL produced by incubating apo‐*Sc*BPL with 500 μM biotin, 1 mM MgCl_2_, and 1 mM ATP on ice for at least 1 h prior to buffer exchange into 100 mM ammonium acetate pH 6.9 using Micro Bio‐Spin P‐6 Gel Columns (Bio‐Rad) (three sequentially). *β*‐lactoglobulin, avidin, albumin, concanavilin A, and alcohol dehydrogenase were used as collision cross section (CCS) calibrants^[^ ^37^ ^]^ and prepared as previously outlined.^[^ ^24^ ^]^ MS analysis was completed using a Synapt G2 S High Definition Mass Spectrometer (HDMS) (Waters Corporation) with samples introduced in the positive ion mode from a nano‐electrospray ionization (nESI) source. The positive potential was applied to the sample along a platinum wire inserted into a glass capillary that was prepared in‐house. Parameters were optimized to maintain noncovalent interactions and native protein conformations, and included capillary voltage 1.01.6 kV; sampling cone 50 V; source temperature 40 °C; trap collision energy 4 V; transfer collision energy 2 V; trap gas flow 2 mL min^−1^; and backing pressure 3.67 mbar. The specific ion mobility parameters included IM cell wave height 35 V; IM cell wave velocity 400 m s^−1^; transfer t‐wave height 0 V; transfer t‐wave velocity 191 m sec^−1^. Data analysis was performed using MassLynx (Waters) with manual peak finding. Drift times were extracted using MassLynx V4.1 and DriftScope 2.8 (Waters Corporation) for calculation of CCS values according to published calculations^[^ ^61^ ^]^ using Microsoft Excel.

##### Collision Induced Unfolding‐Mass Spectrometry (CIU‐MS)

CIU‐MS experiments were completed using previously reported protocols^[^ ^24^ ^]^ with ORIGAMI^MS^ and ORIGAMI^ANALYSE^ utilized for data collection and analysis respectively.^[^ ^62^ ^]^ Data intensities were normalized using the standard procedures in ORIGAMI^ANALYSE^, whereby intensities are normalized to the most abundant peak. CIU heatmaps and heatmap overlays comparing apo‐ and holo‐*Sc*BPL unfolding were produced by ORIGAMI^ANALYSE^ for qualitative comparisons.

##### Circular Dichroism (CD)

Circular dichroism of apo‐ and holo‐*Sc*BPL were completed as previously reported;^[^ ^24^ ^]^ however, holo‐*Sc*BPL was formed in this case by buffer exchanging apo‐*Sc*BPL using Amicon Ultra‐0.5 MWCO 10,000 centrifugal filter units into 10 mM ammonium acetate pH 6.95 with 50 μM biotin, 100 μM MgCl_2_, and 100 μM ATP.

##### Solution Thermal Denaturation Assays to Measure Melting Temperatures

Thermal denaturation assays were completed as previously reported;^[^ ^24^ ^]^ however, holo‐*Sc*BPL was formed by incubating 10 μM apo‐*Sc*BPL with 50 μM biotin, 100 μM MgCl_2_, and 100 μM ATP on ice for 30 min. A 10‐fold dilution into the thermal denaturation reaction resulted in final concentrations 5 μM biotin, 10 μM MgCl_2_ and 10 μM ATP in the holo‐*Sc*BPL reactions.

##### Hydrogen Deuterium Exchange Mass Spectrometry (HDX‐MS)

Apo‐ and holo‐*Sc*BPL were prepared via buffer exchange as for native IM‐MS and diluted to 34 μM. However, the holo‐*Sc*BPL sample was produced by incubation with ligands after buffer exchange (200 μM biotin, 500 μM MgCl_2_ and 500 μM ATP) so that excess ligand was present during the HDX labeling reaction. Deuterium labeling and quenching reactions were performed using the automatic CTC PAL sample manager (LEAP Technologies). *Sc*BPL was diluted ≈15‐fold in 10 mM potassium phosphate in 99.99% deuterium oxide pH 6.6 (pD 7.0) and incubated at 20 °C for varying time points of 30 s, 1 min, 10 min, 30 min, 1 h and 4 h. A time point of 0 was included by diluting *Sc*BPL in 10 mM potassium phosphate in H_2_O pH 7.0. The labeling reactions were quenched by the addition of an equal volume of pre‐cooled 100 mM potassium phosphate pH 2.5. The labeling reactions for all time points were completed in triplicate. The quenched reaction was injected (95 μL) into a nanoACQUITY UPLC system with HDX technology (Waters Corporation). Protein digestion was performed utilizing an online Enzymate immobilized BEH pepsin column (2.1 × 30 mm, Waters) at 20 °C for 1 min with a flow rate of 150 μL min^−1^. Peptide separation was achieved on a C18 column (Waters Corporation Acquity UPLC BEH C18 1.7 μm, 1.0 × 10 mm) at 40 μL min^−1^ flow over 16 min with the following gradients: 0 min, 5% B; 7 min, 35% B; 8 min, 85% B; 11 min, 5% B; 12 min, 95% B; 13 min, 5% B; 14 min, 95% B; and 15 min, 5% B (mobile phases: A, water + 0.1% formic acid; and B, acetonitrile + 0.1% formic acid). Mass spectra were acquired on a SYNAPT G2‐S HDMS in the positive ion mode with a *m/z* range from 290 to 2500. The mass spectrometer was operated in ToF only mode. LeuEnk peptide was used as the Lock Spray. Nonlabelled peptides were identified and analyzed using ProteinLynx Global Server 3.1 software (Waters), while DynamX 2.0 software was used to compare the deuterium uptake‐labeling rate. HDX data were mapped onto the AlphaFold protein model for *Sc*BPL (obtained from the freely available AlphaFold Protein Structure Database, AlphaFold structure reference AF‐P48445‐F1‐v4, UniProt reference P48445) using USCF Chimera.^[^ ^41^ ^,^ ^42^ ^,^ ^63^ ^]^ A sequence alignment confirmed the protein sequence of the AlphaFold structure was identical to the experimental *Sc*BPL (except for the C‐terminal 6‐His tag added for enzyme purification) utilized in this study. Relative HDX uptake across the *Sc*BPL sequence for each analyzed state and time point were converted to b‐factors for each residue within DynamX 2.0, and the *Sc*BPL structural models were colored by b‐factor to represent an indicative measure of deuterium uptake.

## Conflict of Interest

KP is now an AstraZeneca employee; no further work presented here has been conducted using AstraZeneca resources. All other authors declare that they have no conflicts of interest.

## Author Contributions

**Louise M. Sternicki** and **Steven W. Polyak** conceived the project. **Louise M. Sternicki**, **Tara L. Pukala**, **Kamila J. Pacholarz**, and **Kate L. Wegener** collected the data. **Louise M. Sternicki**, **Tara L. Pukala**, **Kamila J. Pacholarz**, **Perdita Barran**, **Kate L. Wegener**, and **Steven W. Polyak** analyzed the results. **Louise M. Sternicki**, **Tara L. Pukala**, **Kamila J. Pacholarz**, **Perdita Barran**, **Grant W. Booker**, **Kate L. Wegener**, and **Steven W. Polyak** assisted with manuscript preparation.

## Supporting information

Supplementary Material

## Data Availability

The data that support the findings of this study are available from the corresponding author upon reasonable request.
