# Supplementary material for: Hydrogen‐Deuterium Exchange Defines Ligand‐Induced Conformational Changes to the Class III Biotin Protein Ligase from Saccharomyces cerevisiae
Source: Chembiochem. 2025 Sep 17;26(20):e202500439. doi: 10.1002/cbic.202500439 (PMC12582158; doi:10.1002/cbic.202500439)
Supplement: Supplementary file 1 — Supplementary Material [file CBIC-26-e202500439-s001.pdf]

SUPPORTING INFORMATION

Hydrogen-Deuterium Exchange Defines Ligand-Induced Conformational Changes to the Class III Biotin Protein Ligase from *Saccharomyces cerevisiae*

Louise M Sternicki[1,5,*], Tara L Pukala[2], Kamila J Pacholarz[3,6], Perdita Barran[3], Grant W Booker[1], Steven W Polyak[1,4,+] and Kate L Wegener[1,4,*,+]

[1] School of Biological Sciences, The University of Adelaide, South Australia 5005, Australia
[2] School of Physics, Chemistry and Earth Sciences, The University of Adelaide, South Australia 5005, Australia
[3] Manchester Institute of Biotechnology, The University of Manchester, Manchester M1 7DN, United Kingdom
[4] Institute for Photonics and Advanced Sensing (IPAS), School of Biological Sciences, The University of Adelaide, South Australia 5005, Australia
[5] Present address: Institute for Biomedicine and Glycomics, Gold Coast, Griffith University, Queensland 4222, Australia

[+] co-senior authors
* co-corresponding authors.

Table of Contents

Figure S1. Biotinylation blot confirms the production of apo-*Sc*BPL. 3

Figure S2. Native MS can distinguish between apo- and holo-treated *Sc*BPL. 4

Figure S3. There is no difference in the secondary structure of apo- (blue) and holo-*Sc*BPL (red), as measured by CD spectroscopy. 5

Figure S4. Collision-induced unfolding MS (CIU-MS) of apo-*Sc*BPL (top) and holo-*Sc*BPL (bottom) for the two most intense charge states, +17 and +16. 6

Figure S5. Solution thermal denaturation assays comparing apo-*Sc*BPL (blue) and holo-*Sc*BPL (red). 7

Figure S6. Structural overlay of AlphaFold predicted *Sc*BPL structure with empirically determined structures of other BPLs… 8

Figure S7. *Sc*BPL does not contain glutamine amidotransferase (GATase) activity as measured by ^{1}H 1D NMR spectroscopy. 9

Figure S8. *Sc*BPL activity is not altered in the presence of glutamine. 10

Figure S9. Sequence coverage of *Sc*BPL by the peptides identified from HDX LC-MS. 11

Figure S10. The HDX results for apo-*Sc*BPL mapped across the sequence of *Sc*BPL. 12

Figure S11. Heat map identifying the difference in deuterium incorporation between apo- and holo-*Sc*BPL across the sequence. 14

Figure S12. Examples of the deuterium uptake rates in apo-*Sc*BPL (blue) compared to holo-*Sc*BPL (green) for specific peptides measured over the four-hour time-course. 15

Figure S13. Sequence alignment of example eukaryotic class III BPL sequences from fungi and other animals. 17

Figure S14. Mapping the *Sc*BPL residues analogous to the human BPL N-terminal domain mutations that cause Multiple Carboxylase Disease (MCD) onto the *Sc*BPL AlphaFold structure. 23

Table S1. Masses of the species observed in the MS spectra of apo- and holo-treated *Sc*BPL. 24

Table S2. Full width half maximum (FWHM) calculated from the IM-MS $^{TW}CCS_{N2}$ distribution data for all charge states (15+ to 18+) for apo-*Sc*BPL and holo-*Sc*BPL. 25

Table S3. Structural similarity, measured by RMSD, between the *Sc*BPL C-terminal catalytic domain model produced by AlphaFold and crystal structures of Class I and II BPLs from other species. 26

Methods: 1D ^{1}H NMR for glutamine amidotransferase activity. 27

Methods: *In vitro* biotinylation assays. 27

References 28

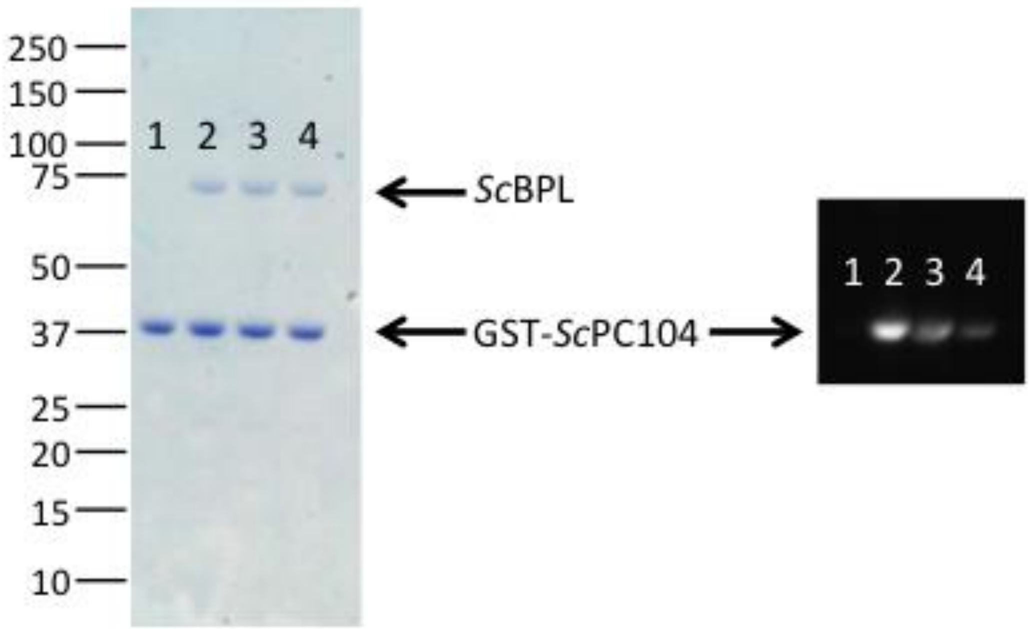

Figure S1. Biotinylation blot confirms the production of apo-*Sc*BPL. Apo-*Sc*BPL was measured for its ability to biotinylate the GST-conjugated biotin domain GST-*Sc*PC104 – the biotin domain from the *S. cerevisiae* pyruvate carboxylase isoform 1. Reactions containing 50 mM Tris pH 8.0, 3 mM ATP, 5.5 mM $MgCl_2$, 0.1 mM DTT, 15 μM GST-*Sc*PC104 and 3 μM *Sc*BPL were incubated in the presence and absence of biotin for 1 hour at 37 °C. Biotinylation was detected utilising a streptavidin-conjugated fluorophore (Alexa488). Biotinylation of GST-*Sc*PC104 by apo-*Sc*BPL in the absence of biotin indicates remaining bound biotin or biotinyl-5′-AMP from expression and purification. Lanes include 1) 15 μM unbiotinylated GST-*Sc*PC104 to measure the intrinsic biotinylation of the GST-*Sc*PC104 protein preparation (no *Sc*BPL or biotin in reaction), 2) 15 μM biotinylated GST-*Sc*PC104 to act as a measure of complete GST-*Sc*PC104 biotinylation (15 μM biotin in reaction to allow complete biotinylation of 15 μM GST-*Sc*PC104), 3) 3 μM biotinylated GST-*Sc*PC104 to measure the biotinylation signal from 3 μM of holo-*Sc*BPL (3 μM biotin in reaction to partially biotinylate the 15 μM GST-*Sc*PC104), and 4) 3 μM apo-*Sc*BPL + 15 μM unbiotinylated GST-*Sc*PC104 to measure the biotinylation from the apo-*Sc*BPL preparation. A faint band present in lane 4 indicates there was some biotin or biotinyl-5-AMP in the apo-*Sc*BPL preparation that could biotinylate the apo-GST-*Sc*PC104 (measured to have no intrinsic biotinylation by lane 1). However, the intensity of this band was minimal compared to the maximal amount of biotinylation possible from 3 μM of holo-*Sc*BPL (lane 3), suggesting only a minor fraction of holo-*Sc*BPL remained that would not interfere significantly with the further techniques employed in the study.

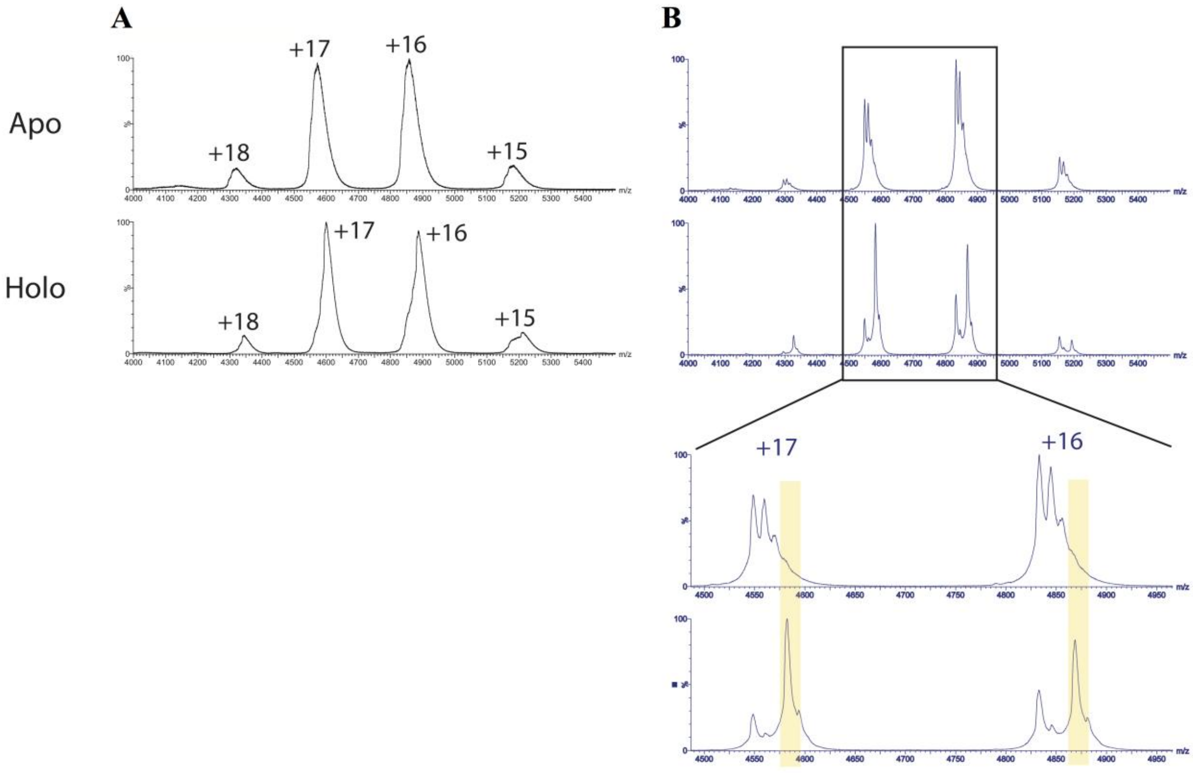

Figure S2. Native MS can distinguish between apo- and holo-treated *Sc*BPL. Spectra at cone voltage of A) 50 V (also shown in Figure 2B) and B) 200 V (blue). The inset in panel B shows a zoomed in view of charge states +17 and +16, with yellow highlights indicating the mass range expected for the holo species.

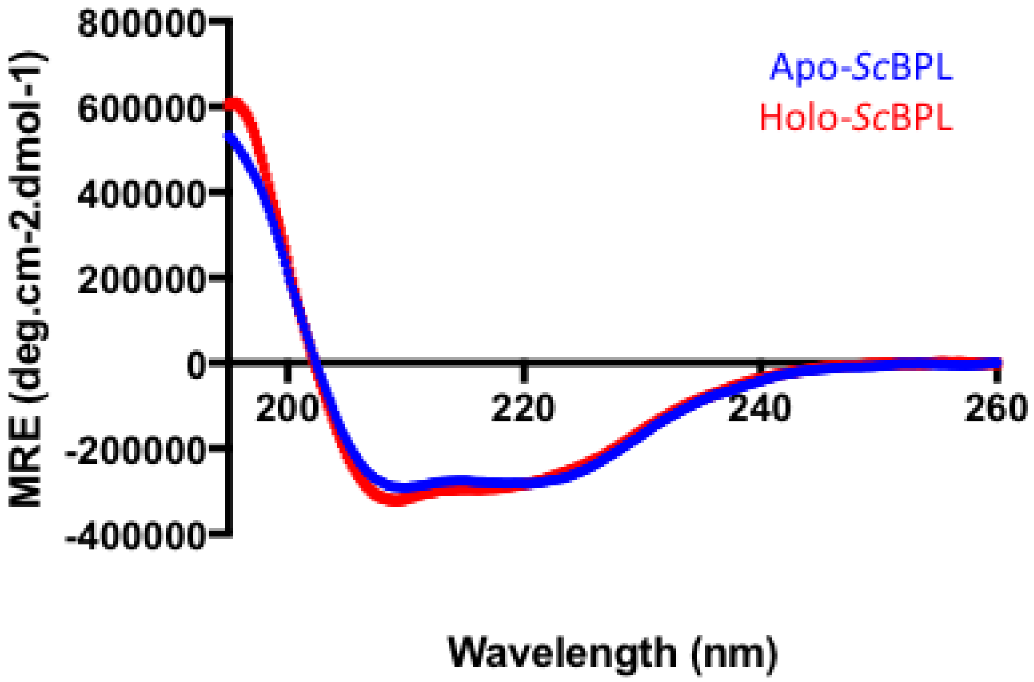

Figure S3. There is no difference in the secondary structure of apo- (blue) and holo-*Sc*BPL (red), as measured by CD spectroscopy. Spectra are the smoothed average of 5 scans corrected for buffer and protein concentration.

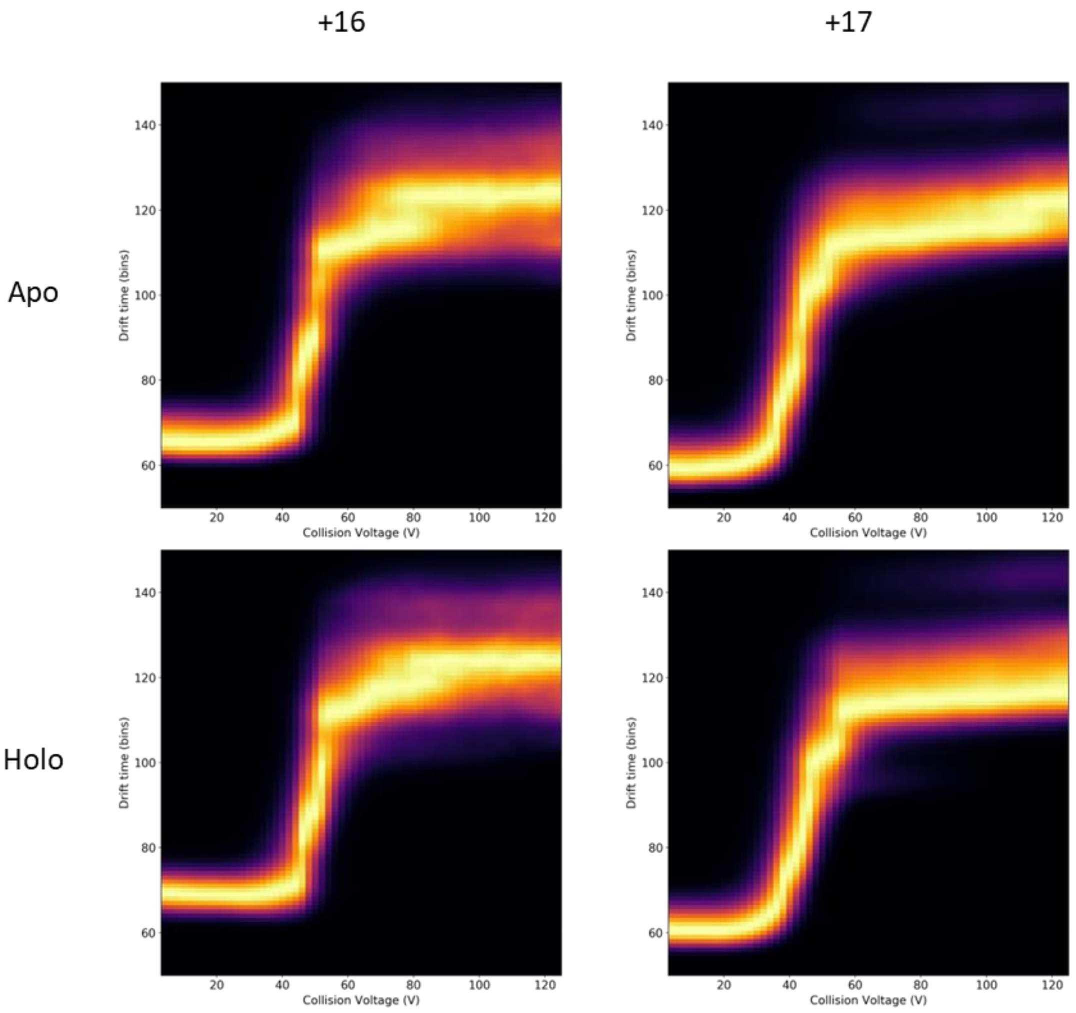

Figure S4. Collision-induced unfolding MS (CIU-MS) of apo-*Sc*BPL (top) and holo-*Sc*BPL (bottom) for the two most intense charge states, +17 and +16. Increasing voltages (x-axis) are applied to the protein to unfold it, causing an increase in protein size and, therefore, drift time (y-axis).

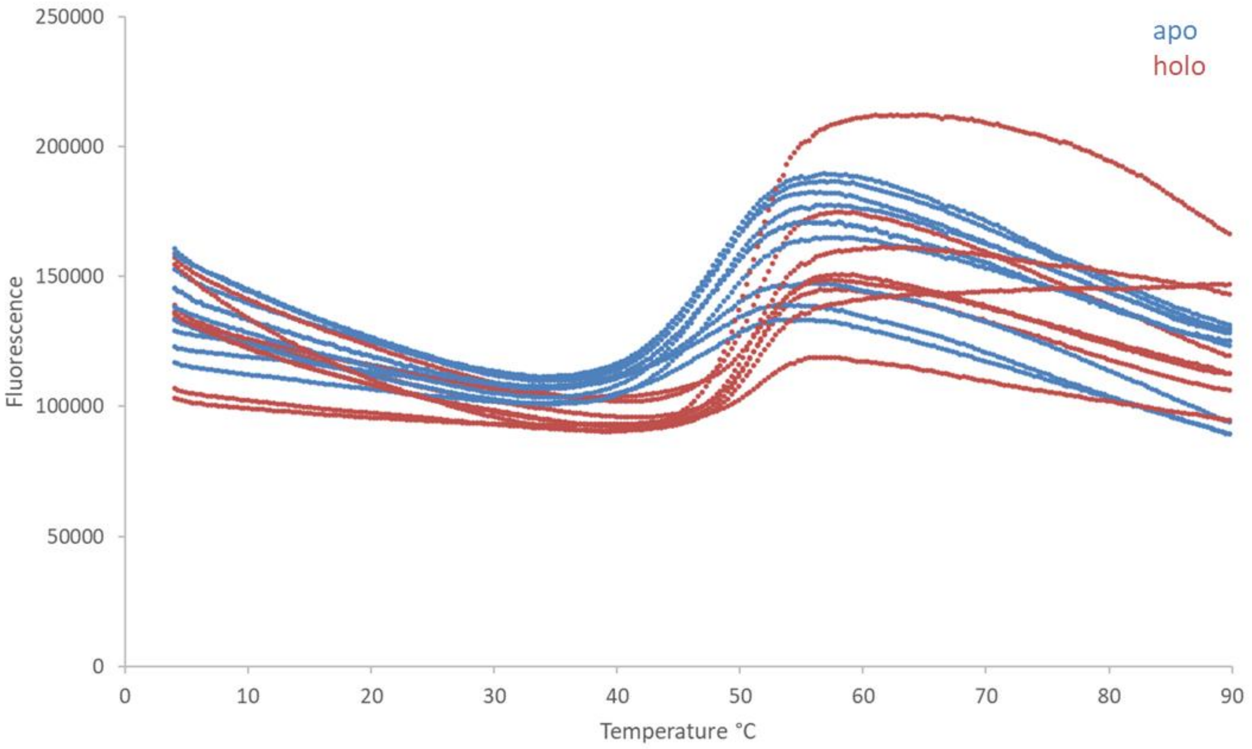

Figure S5. Solution thermal denaturation assays comparing apo-*Sc*BPL (blue) and holo-*Sc*BPL (red). Raw data for all replicates are shown (three technical replicates for three independent biological assay replicates).

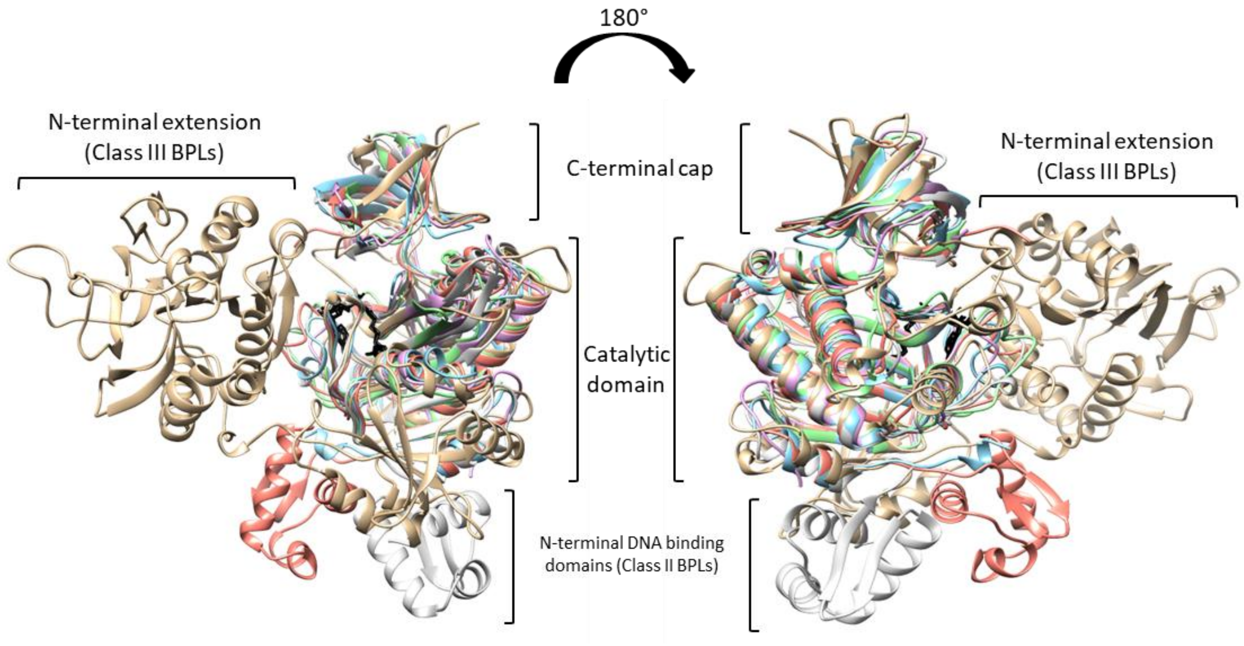

Figure S6. Structural overlay of AlphaFold predicted *Sc*BPL structure (beige, AF-P48445-F1-v4, UniProt P48445)[1-2]) with empirically determined structures of other BPLs, including Class I BPLs from *M. tuberculosis* (blue, PDB: 4OP0[3]), *P. horikoshii* (purple, PDB: 1WPY[4]) and *A. aquifex* (green, PDB: 2EAY[5]) and Class II BPLs from *E. coli* (red, PDB: 2EWN[6]) and *S. aureus* (grey, PDB: 3RIR[7]).

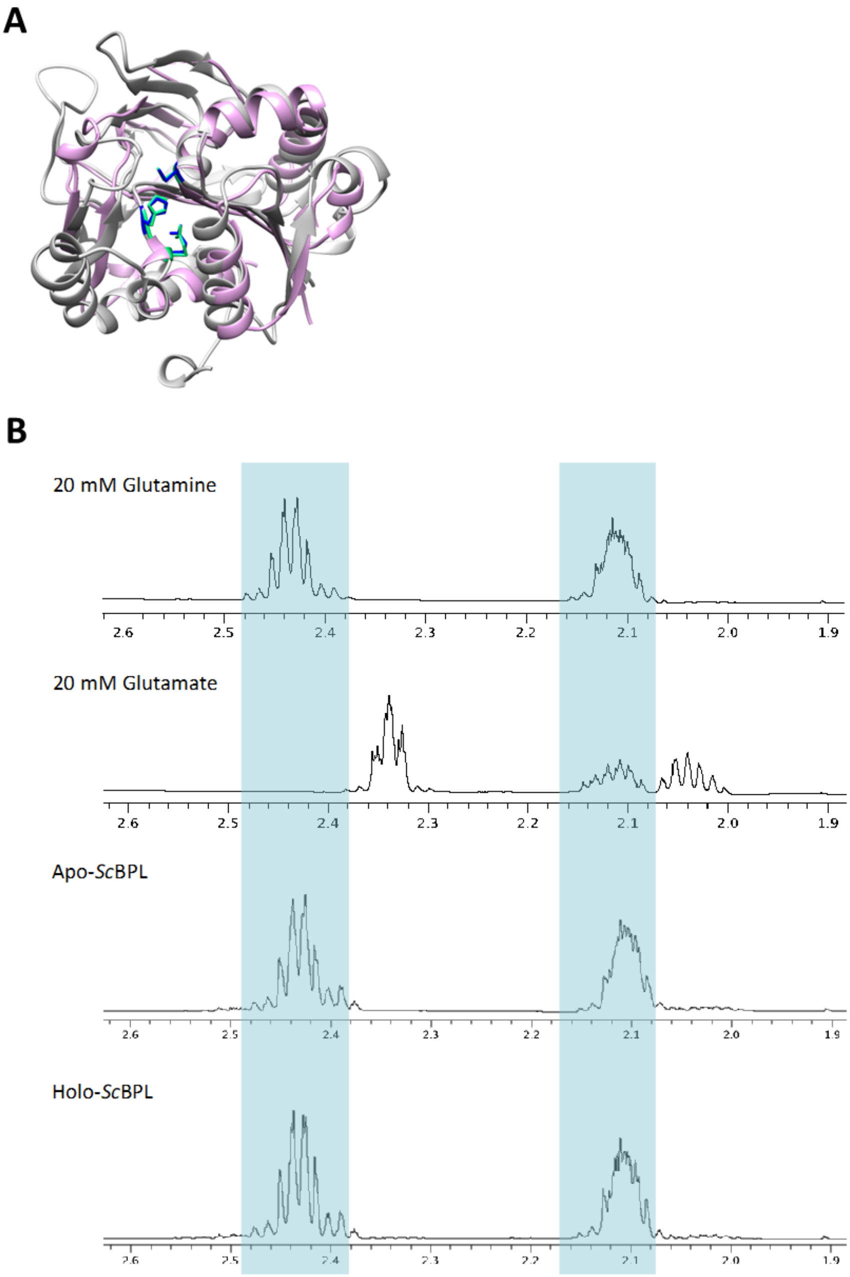

Figure S7. *Sc*BPL does not contain glutamine amidotransferase (GATase) activity as measured by ^{1}H 1D NMR spectroscopy. A) Structural overlay demonstrating the homology of the *Sc*BPL N-terminal domain (AlphaFold model ((AF-P48445-F1-v4, UniProt P48445)[1-2]), grey) with glutamine amidotransferases (GATase) (shown here as an example is the GATase from the *Thermotoga maritima* pyridoxal 5′-phosphate holoenzyme (purple, PDB: 2ISS),[8] the most homologous GATase from Phyre homology modelling[9]). The GATase catalytic triad of residues (cysteine, histidine and glutamic acid, green) were conserved and correctly positioned in the AlphaFold model of the *Sc*BPL N-terminal domain (C89, H215, E217, blue). B) Both apo- and holo-*Sc*BPL (biotin and MgATP added) were incubated with glutamine and the reaction monitored by 1D ^{1}H NMR over 4 days for conversion to glutamate to indicate GATase activity. Controls of 20 mM glutamine and glutamate were used for chemical shift comparison.

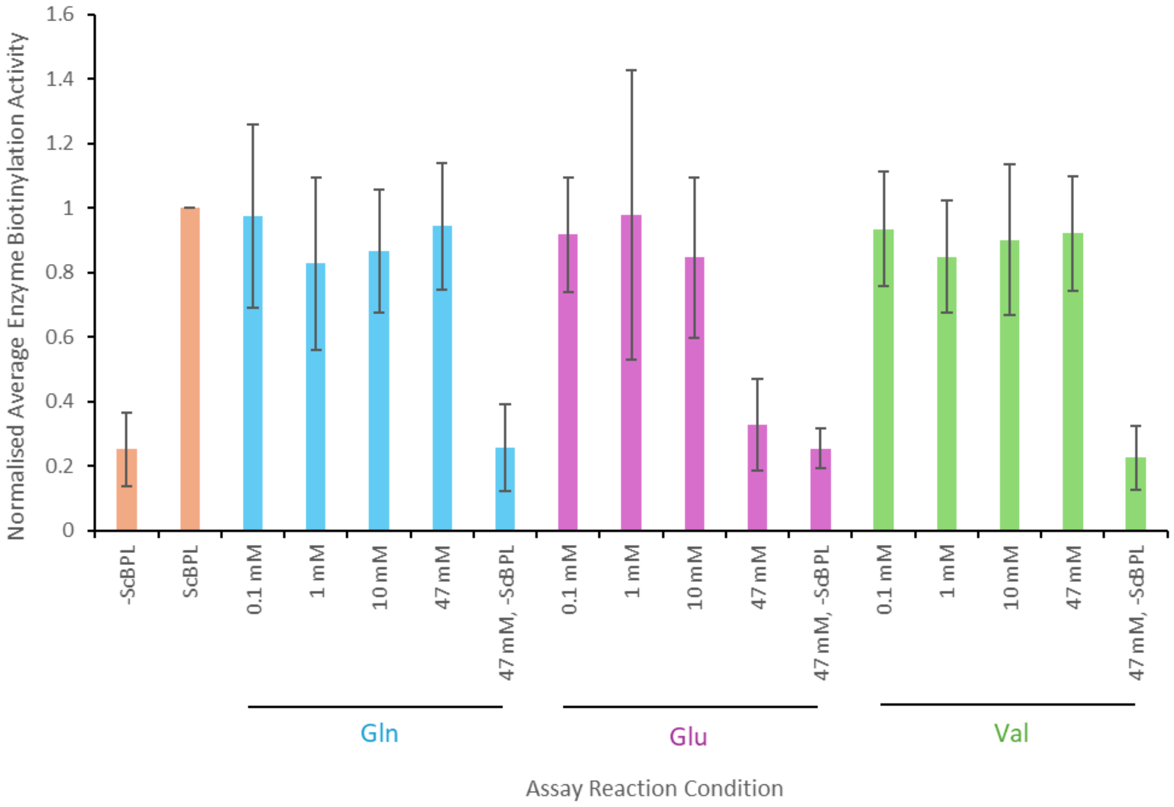

Figure S8. *Sc*BPL activity is not altered in the presence of glutamine (Glu, blue, polar uncharged amino acid). Comparison with the addition of glutamic acid (Glu, purple) and valine (Val, green) as controls representing polar, charged and nonpolar amino acids are included. No *Sc*BPL (-*Sc*BPL) controls are also shown. Assay data was normalised to *Sc*BPL activity in the absence of added amino acids to control for activity count variance between assays. N=4.

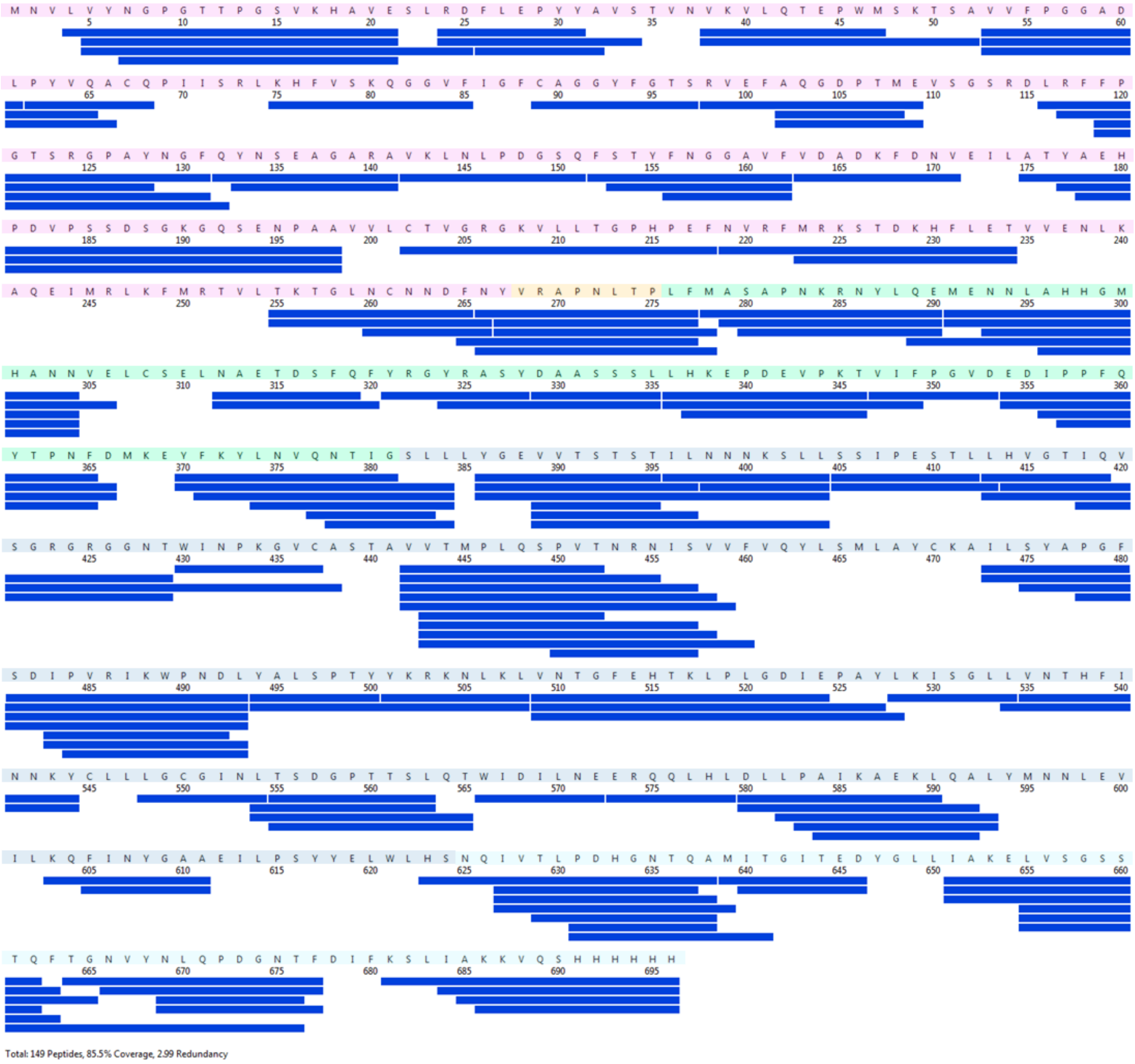

Figure S9. Sequence coverage of *Sc*BPL by the peptides identified from HDX LC-MS. A total of 149 peptides were identified, resulting in 85.5% coverage of *Sc*BPL with a redundancy of 2.99. *Sc*BPL domain architecture is annotated via shading of the *Sc*BPL sequence (pink: N-terminal domain, orange: linker, green: extended catalytic domain region, blue: catalytic domain, light blue: C-terminal cap).

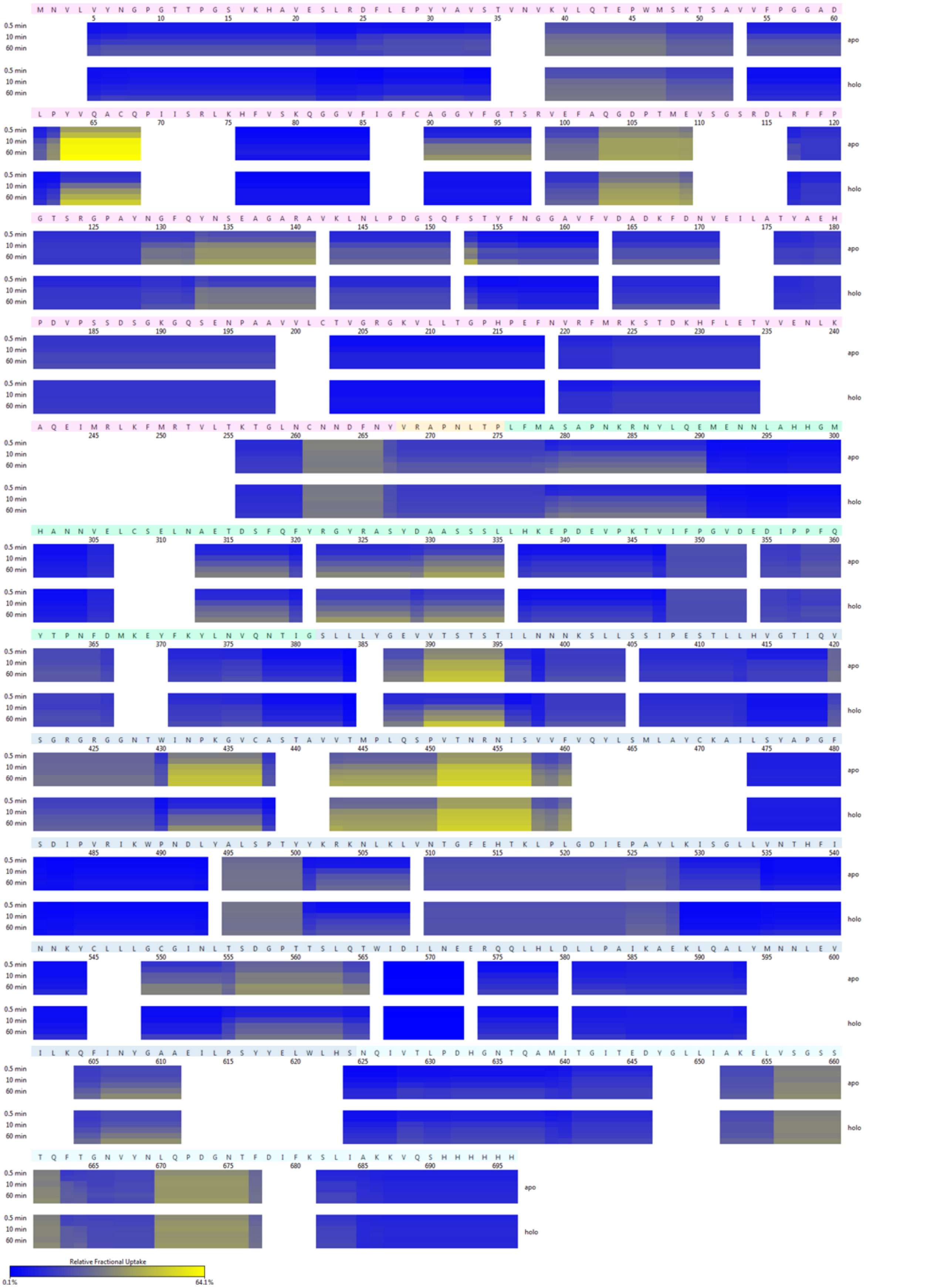

Figure S10. The HDX results for apo-*Sc*BPL mapped across the sequence of *Sc*BPL. Yellow colouring signifies the incorporation of deuterium over the various time points. *Sc*BPL domain

architecture is annotated via shading of the *Sc*BPL sequence (pink: N-terminal domain, orange: linker, green: extended catalytic domain region, blue: catalytic domain, light blue: C-terminal cap).

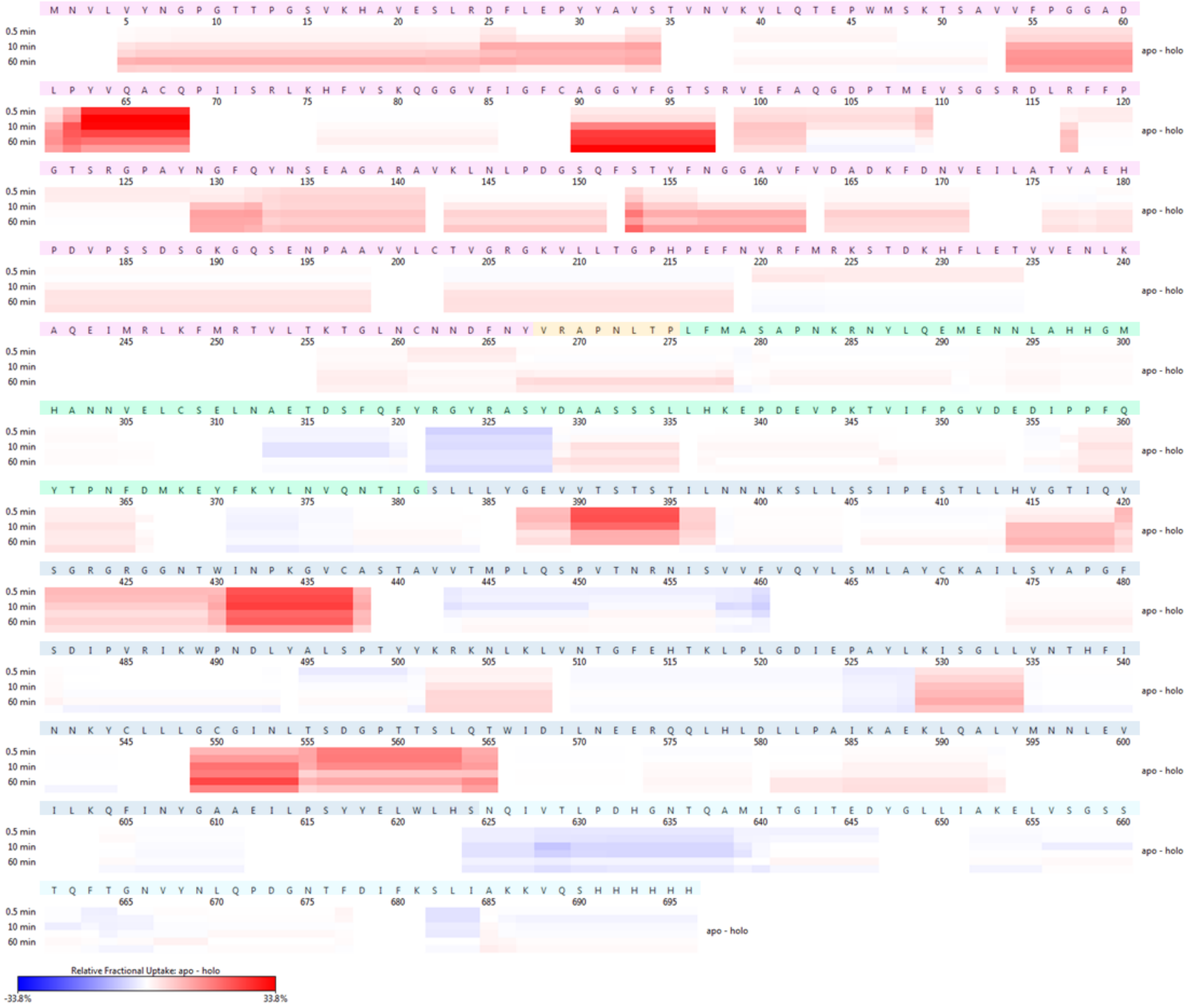

Figure S11. Heat map identifying the difference in deuterium incorporation between apo- and holo-*Sc*BPL across the sequence. Red colouring indicates greater deuterium incorporation for apo-*Sc*BPL, whilst blue indicates more deuterium uptake in holo-*Sc*BPL. *Sc*BPL domain architecture is annotated via shading of the *Sc*BPL sequence (pink: N-terminal domain, orange: linker, green: extended catalytic domain region, blue: catalytic domain, light blue: C-terminal cap).

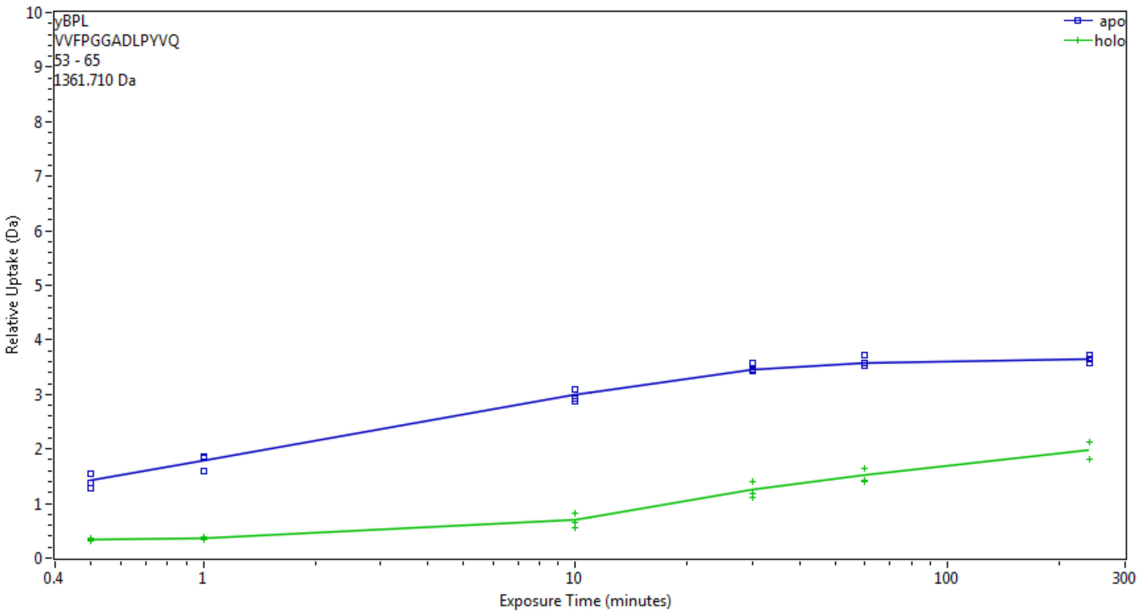
yBPL
VVFPGGADLPYVQ
53 - 65
1361.710 Da
apo
holo
Relative Uptake (Da)
Exposure Time (minutes)
0
1
2
3
4
5
6
7
8
9
10
0.4
1
10
100
300

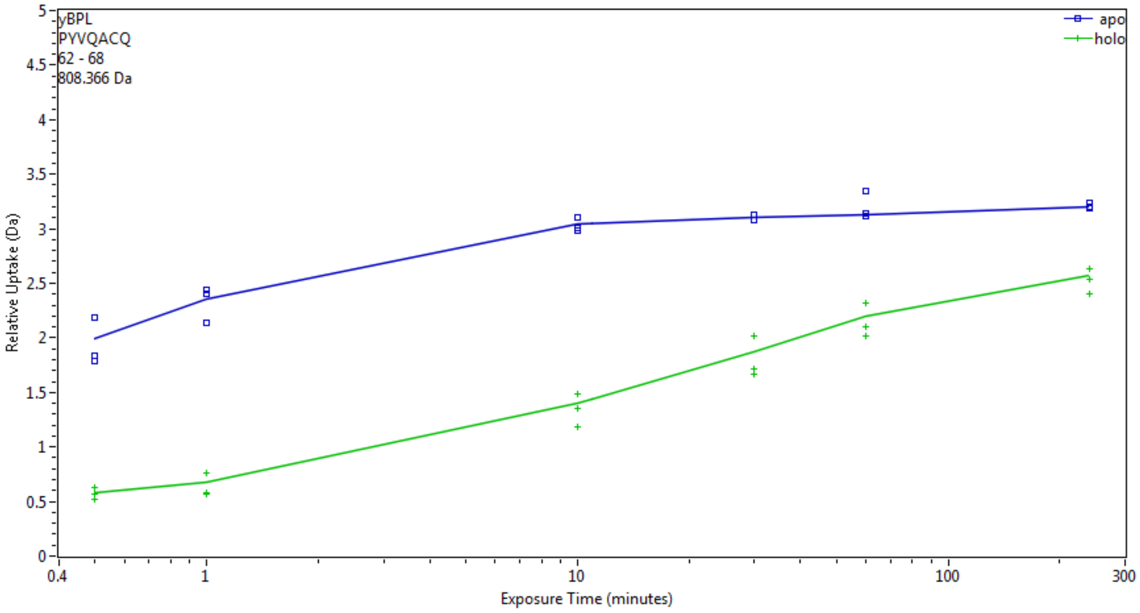
yBPL
PYVQACQ
62 - 68
808.366 Da
apo
holo
Relative Uptake (Da)
Exposure Time (minutes)
0
0.5
1
1.5
2
2.5
3
3.5
4
4.5
5
0.4
1
10
100
300

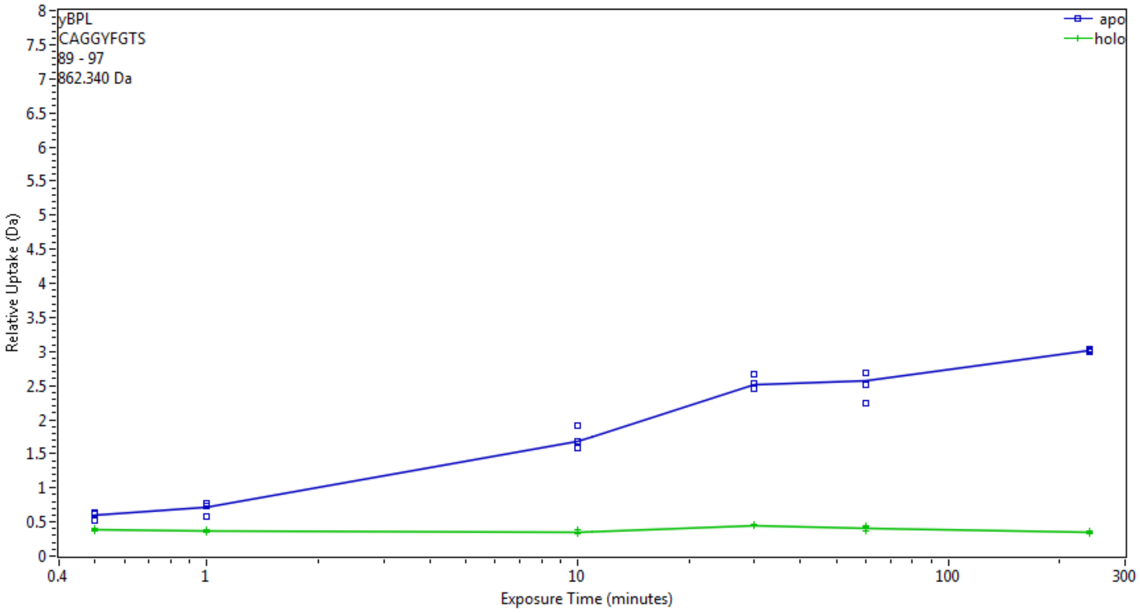
yBPL
CAGGYFGTS
89 - 97
862.340 Da
apo
holo
Relative Uptake (Da)
Exposure Time (minutes)
0
0.5
1
1.5
2
2.5
3
3.5
4
4.5
5
5.5
6
6.5
7
7.5
8
0.4
1
10
100
300

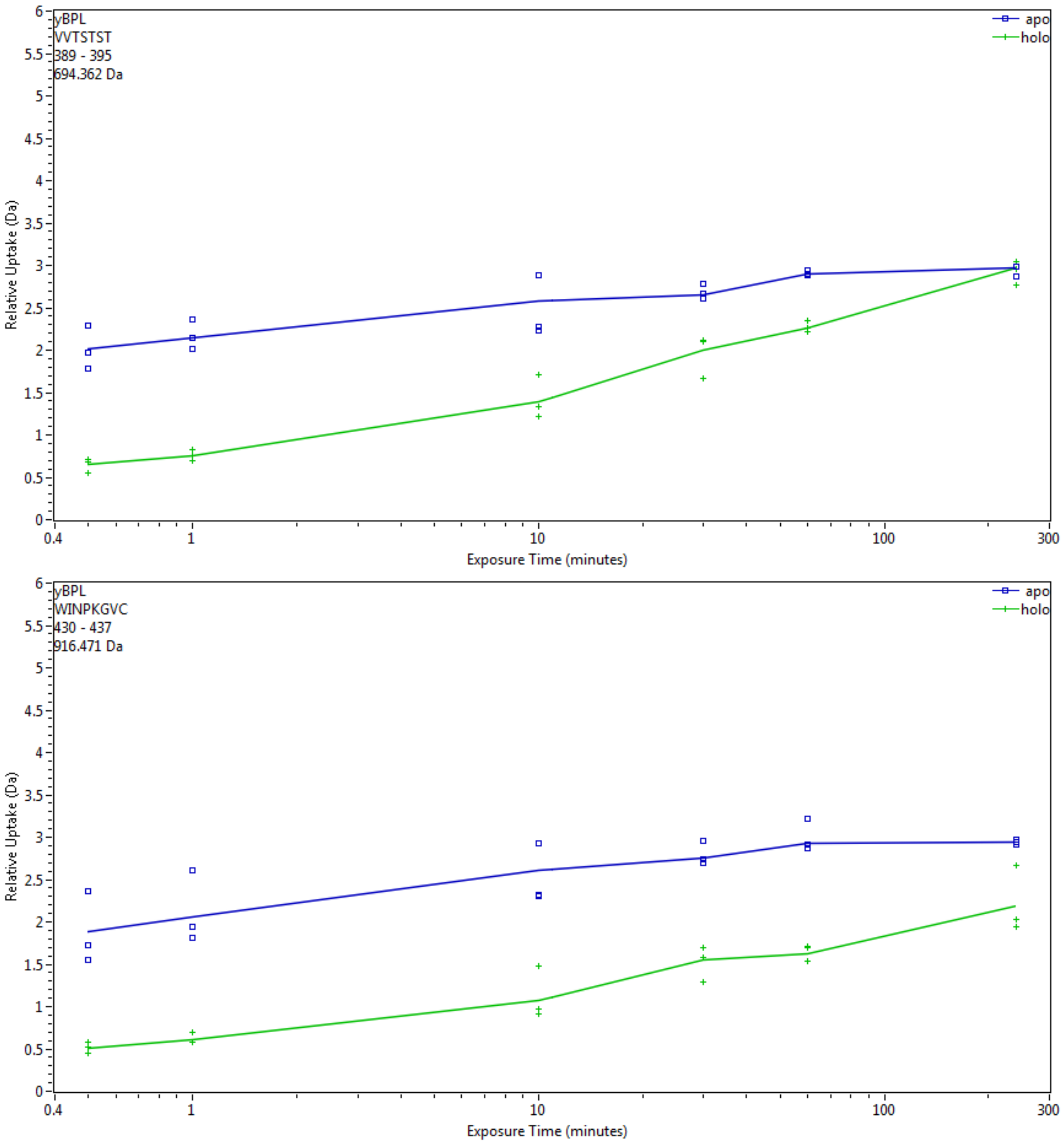

Figure S12. Examples of the deuterium uptake rates in apo-*Sc*BPL (blue) compared to holo-*Sc*BPL (green) for specific peptides measured over the four-hour time-course. The corresponding residue numbering for the peptide position within *Sc*BPL is annotated.

```
C.neoformans        ------------------------------------------------------------   0
S.pombe             ------------------------------------------------------------   0
S.cerevisiae        ------------------------------------------------------------   0
C.albicans          ------------------------------------------------------------   0
Z.tritici           ------------------------------------------------------------   0
A.fumigatus         ------------------------------------------------------------   0
B.cinerea           ------------------------------------------------------------   0
C.elegans           ------------------------------------------------------------   0
D.melanogaster      MLTLYYVSATVLQSWRIQKACSKIAEHLAQPSSIAFYTLQSGSDDGFDPALASSELCCNR   60
M.Mulatta           ------------------------------------------------------------   0
H.sapiens           ------------------------------------------------------------   0
M.musculus          ------------------------------------------------------------   0
R.norvegicus        ------------------------------------------------------------   0


C.neoformans        ------------------------------------------------------------   0
S.pombe             ------------------------------------------------------------   0
S.cerevisiae        ------------------------------------------------------------   0
C.albicans          ------------------------------------------------------------   0
Z.tritici           ------------------------------------------------------------   0
A.fumigatus         ------------------------------------------------------------   0
B.cinerea           ------------------------------------------------------------   0
C.elegans           ------------------------------------------------------------   0
D.melanogaster      NAAKVTDILWLHANQRGCCLRPLQTLHITPWISFPPAPSLLPFSYAADTLTPASTPTEAD   120
M.Mulatta           ------------------------------------------------------------   0
H.sapiens           ------------------------------------------------------------   0
M.musculus          ------------------------------------------------------------   0
R.norvegicus        ------------------------------------------------------------   0


C.neoformans        ------------------------------------------------------------   0
S.pombe             ------------------------------------------------------------   0
S.cerevisiae        ------------------------------------------------------------   0
C.albicans          ------------------------------------------------------------   0
Z.tritici           ------------------------------------------------------------   0
A.fumigatus         ------------------------------------------------------------   0
B.cinerea           ------------------------------------------------------------   0
C.elegans           ------------------------------------------------------------   0
D.melanogaster      VPRQQRVSLSAQGEERMQLLLEADIEPLQRPSSEDTSAVRLEDYGKLIAWKIDSHLAVLI   180
M.Mulatta           ------------------------------------------------------------   0
H.sapiens           ------------------------------------------------------------   0
M.musculus          ------------------------------------------------------------   0
R.norvegicus        ------------------------------------------------------------   0


C.neoformans        ------------------------------------------------------------   0
S.pombe             ------------------------------------------------------------   0
S.cerevisiae        ------------------------------------------------------------   0
C.albicans          ------------------------------------------------------------   0
Z.tritici           ------------------------------------------------------------   0
A.fumigatus         ------------------------------------------------------------   0
B.cinerea           ------------------------------------------------------------   0
C.elegans           ------------------------------------------------------------   0
D.melanogaster      ETDVEHFTKLLITTFLRNNLCINDQLPLLRIESVQREGDPQPFELLAKHLKRQSRISVGL   240
M.Mulatta           ------------------------------------------------------------   0
H.sapiens           ------------------------------------------------------------   0
M.musculus          ------------------------------------------------------------   0
R.norvegicus        ------------------------------------------------------------   0
```

```
C.neoformans        ------------------------------------------------------------    0
S.pombe             ------------------------------------------------------------    0
S.cerevisiae        ------------------------------------------------------------    0
C.albicans          ------------------------------------------------------------    0
Z.tritici           ------------------------------------------------------------    0
A.fumigatus         ------------------------------------------------------------    0
B.cinerea           ------------------------------------------------------------    0
C.elegans           ------------------------------------------------------------    0
D.melanogaster      DKDGWKKHMEDLRA--VGVLAHQATEFEYQRNRSEGRTKSDPTTHDQRPTSELLAKAVAV    298
M.Mulatta           --------MEDRLHMDNGLVPQKIMSVRLQ----------DSTLKEVKD--QASNKQAQI    40
H.sapiens           --------MEDRLHMDNGLVPQKIVSVHLQ----------DSTLKEVKD--QVSNKQAQI    40
M.musculus          --------MEDRLQMDNGLIAQKIVSVHLK----------DPALKELG---KASDKQVQG    39
R.norvegicus        --------MEDRLQMDNGLIAQKIVSVHLK----------DPALKELS---KASNKQVQA    39

C.neoformans        ------------------------------------------------------------    0
S.pombe             ------------------------------------------------------------    0
S.cerevisiae        ------------------------------------------------------------    0
C.albicans          ------------------------------------------------------------    0
Z.tritici           ------------------------------------------------------------    0
A.fumigatus         ------------------------------------------------------------    0
B.cinerea           ------------------------------------------------------------    0
C.elegans           -------------------------------MNFPRVISNARHIALLFTGDRG-------    22
D.melanogaster      VEPTNKAY--LSPART-TEASLKAEAKGTSTKPFPTKSDAKPATLAKSEGTPATSKEDSK    355
M.Mulatta           LEPRPEPSLEIKPEQDGMEHVGRDDPKALGEEPK-QRRGSASGSEPAGDSDRG-------    92
H.sapiens           LEPKPEPSLEIKPEQDGMEHVGRDDPKALGEEPK-QRRGSASGSEPAGDSDRG-------    92
M.musculus          PPPGPEASPEAQPAQGVMEHAGQGDCKAAGEGPSPRRRGCAPESEPAADGDPG-------    92
R.norvegicus        PLPSPEASLEAQPAQGVMEQAGQGDWKAAGEGPSPQRRGCAPVSESAADGDPG-------    92

C.neoformans        ------------------------------------------------------------    0
S.pombe             ------------------------------------------------------------    0
S.cerevisiae        ------------------------------------------------------------    0
C.albicans          ------------------------------------------------------------    0
Z.tritici           ------------------------------------------------------------    0
A.fumigatus         ------------------------------------------------------------    0
B.cinerea           ------------------------------------------------------------    0
C.elegans           --------------------------------RSV---------------SPS----FEY    31
D.melanogaster      LTPTKGALKTSELEKLAAVQAAQQQKKEAVQVSPVKAAFLAKPPMLSKHDEPDKASDQPT    415
M.Mulatta           ----GGPIEHYHLH---LSSCHECLELENSTIESVKFA--------SAENIPD----LPY    133
H.sapiens           ----GGPVEHYHLH---LSSCHECLELENSTIESVKFA--------SAENIPD----LPY    133
M.musculus          ----LSSPELCQLH---LSICHECLELENSTIDSVRSA--------SAENIPD----LPC    133
R.norvegicus        ----QGSTELCQLH---LSSCHECLELENSTIESVRCA--------SAENIPD----LPH    133

C.neoformans        ---------------------------------------MPGPTPSAHQVLV--------    13
S.pombe             -----------------------------------------------MNVLI--------    5
S.cerevisiae        -----------------------------------------------MNVLV--------    5
C.albicans          -----------------------------------------------MNVLV--------    5
Z.tritici           -----------------------------------------MAPTKRLNVLI--------    11
A.fumigatus         -----------------------------------MTATTPNLTGKKLNVLVYSGSLTQS    25
B.cinerea           ------------------------------------------MVARKMDVLV--------    10
C.elegans           YQNRSLRGFTSAANPHRVATRNSTVRQVPLYQFLTSRSSSFKAMCKPNSVLV--------    83
D.melanogaster      KPRKSFEAVKPLNSPP-------SSRAAPSRPV-LQRNKDSLQDAKPLNVLV--------    459
M.Mulatta           DYSSSLESVADE--------------TSPER-----EGRRVNLTGKAPNILL--------    166
H.sapiens           DYSSSLESVADE--------------TSPER-----EGRRVNLTGKAPNILL--------    166
M.musculus          DH-SGVEGAAGE--------------LCPER-----KGKRVNISGKAPNILL--------    165
R.norvegicus        DCSSSVEGTAGE--------------LCPER-----KGKRVNISGKAPNILL--------    166
                                                                    .:*:
```

```
C.neoformans        ------------YAGPGVSPL-----SLSHTLLTLRLLLLPHYTVQPAAPDLLAHQPWEP    56
S.pombe             ------------YNGNGASKI-----CLLRTFQSLLPFVVPLYAMRFVDASTLEKEPWPA    48
S.cerevisiae        ------------YNGPGTTPG-----SVKHAVESLRDFLEPYYAVSTVNVKVLQTEPWMS    48
C.albicans          ------------YSGPGTTTE-----GVKHCLETLRLHLGSYYAVLPVNETVLLNEPWMR    48
Z.tritici           ------------YTGTGASLS-----SVRHATWSLRRLLGPHYAVLTVSADQILKEPWSA    54
A.fumigatus         AWRSTSLTSSPPSPGNGSTVE-----SVRHCLYTLRRLLAPHYAVIPVTADMLIKEPWTL    80
B.cinerea           ------------YSGNGSTIE-----SVRHCLYTLRRLLSPIYAVIPVSDAVILKEPWTA    53
C.elegans           ------------YTGGNDSL----YSDIRHRLSFLL--PPDEITVFNVSIQALKKQPWAE    125
D.melanogaster      ------------YSDSASARE-----STLATLQQLL--ERNVYTIYPLMPQQAAQKYWTE    500
M.Mulatta           ------------YVGSDSQEALGRFHEVRSVLADCV--DIDSYVLYHLLEDSALRDPWTD    212
H.sapiens           ------------YVGSDSQEALGRFHEVRSVLADCV--DIDSYILYHLLEDSALRDPWTD    212
M.musculus          ------------YVGSGSEEALGRLQQVRSVLTDCV--DTDSYTLYHLLEDSALRDPWSD    211
R.norvegicus        ------------YVGSGSEEALGQLQQVRSVLTDCV--DTDSYTLYHLLEDSALRDPWPD    212
                                  .                             :          . *

C.neoformans        SCALLVVPGGRDLPYVDELTDKRPVTTRIKEYVQQGGRFLGICAGAYFASAEVR------    110
S.pombe             STALLVMPGGRDMGYCSSFNET--IYRKITDFVKRGGAYLGLCAGGYFGSAVVD------    100
S.cerevisiae        KTSAVVFPGGADLPYVQACQPI--ISRLKHFVSKQGGVFIGFCAGGYFGTSRVE------    100
C.albicans          KTSLLVIPGGADLPYCNVLDGN--GTRKISKYVKQGGKFLGLCAGGYFGSARCE------    100
Z.tritici           TCALLVMPGGADSGYCRTLNGD--GNRKIKRYVQLGGKYLGLCAGGYYGCARCE------    106
A.fumigatus         TCALLVIPGGADLGYCRSLNGT--GNRRIEQFVKRGGAYLGFCAGGYYGSKRCE------    132
B.cinerea           SCALLVFPGGADQGYCRSLNGE--GNRRISQYVRRGGAYLGFCAGGYYGTSRCE------    105
C.elegans           KSTVCVILASTND-----LDDE--AWEKIQAYFNQNGKIIFVCQNKLLASITGCDSSKAN    178
D.melanogaster      QTALLVV-CG--S-----VAHG--IGQILVDYFLQGGKVLSLCSDILHFVLPNYRTAEVR    550
M.Mulatta           NCLLLVI-ATRES-----IPED--LYQKFMAYLSQGGKVLGLSSSFTFGGFQVTSKGALR    264
H.sapiens           NCLLLVI-ATRES-----IPED--LYQKFMAYLSQGGKVLGLSSSFTFGGFQVTSKGALH    264
M.musculus          NCLLLVI-ASRDP-----IPKD--IQHKFMAYLSQGGKVLGLSSPFTLGGFRVTRRDVLR    263
R.norvegicus        NCLLLVI-ASRDP-----IPKG--IHHRFMAYLSQGGKVLGLSSSFTFGDLRVARRDVLR    264
                         *.                            .*  : ..

C.neoformans        -------FDVGG----GMEVAGKRDLAFFPGPSRGPVFQGFQYASESGSRAVV--LDLES    157
S.pombe             -------FRMPDS---DLNVVGKRKLQFFPGTCAGPTFPGFTYDSEDGARRAS--IIVDG    148
S.cerevisiae        -------FAQGDP---TMEVSGSRDLRFFPGTSRGPAYNGFQYNSEAGARAVK--LNLPD    148
C.albicans          -------FEVGNP---TMEVTGPRELGFFPGTAKGCAFKGFKYESRTGARAVK--LSVNT    148
Z.tritici           -------FEVGKK---GMEVVGDRELAFFPGICRGLAYPGFVYASEAGARAVE--ILVNK    154
A.fumigatus         -------FEVGDK---TMEVVGDRELAFFPGICRGGAFPGFLYHSEVGARAAD--LKFSK    180
B.cinerea           -------FEVGNK---QLEVVASRELQFYPGTCRGCAYKGFVYHSEAGAKAAE--VKIE-    152
C.elegans           --ASILRFAFGSQNN-KLKDTNKEFVKFLEKNMK----KL--------PKST--------    215
D.melanogaster      EH-ELVQFSYDKW--QRVKMMHHIF-CYQPSPVK----KHFSTDSEESTKSHSRKPSMEL    602
M.Mulatta           KTVQNLVFSKADQSEVKLSVLSSGC-RYQEGPVR-----------------------LSP    300
H.sapiens           KTVQNLVFSKADQSEVKLSVLSSGC-RYQEGPVR-----------------------LSP    300
M.musculus          NTVQNLVFSKADGTEVRLSVLSSGY-VYEEGPS--------------------------L    296
R.norvegicus        NTAQNLVFSKADGSEVRLSVLSSGY-VYEEGPS--------------------------L    297
                           *         :.        :

C.neoformans        SKLE-----TLNHIYYNGGGHFIFSSPP-PPNVQILARFQETSSDPS-------EQQLVA    204
S.pombe             M----QSSPVHTHIYFNGGGSFLETENYSN--VKVVARYQETD-----------FEKSAA    191
S.cerevisiae        G--------SQFSTYFNGGAVFVDADKFDN--VEILATYAEHPDVPSSDSGKGQSENPAA    198
C.albicans          AALP-G-CASHIYNYYDGGAVFANAEKYKD--VEILARYDDKTDIVD--------LEKAA    196
Z.tritici           GAL-SGVVVGSFRSYYNGGGTFVDAEKMEETGVEVLASFAEKLAVES-------GEAKAA    206
A.fumigatus         DVLQDGVVPEGFKCYYNGGGVFVDAPLYADRGVEVLASYTEELNVDP-------GAGAAA    233
B.cinerea           GSLQSGNAPQSFKSYYNGGGVFVDAGKFKDQGVEILATYVHPVDVDG-------GEDPAA    205
C.elegans           -AIN---------------ETF------RSKDVSV-GANFT-V-VL-------KKEPDAP    243
D.melanogaster      KDLA---------------GHS------HNLDVHVLGTEET------------WNTPSLM    629
M.Mulatta           GGLQ---------------GHL------ENDDKDRM----------------------IV    317
H.sapiens           GRLQ---------------GHL------ENEDKDRM----------------------IV    317
M.musculus          GRLQ---------------GHL------ENEDKDKM----------------------IV    313
R.norvegicus        GRLQ---------------GHL------ENEDKDKM----------------------IV    314
```

```
C.neoformans      AVFTQNGKGCTILSSVHPEYPLSDPPASNA--------IAKLDVRP-------SQVEVEM    249
S.pombe           IIYVKVGKGNVVLTGIHPEFSAEGSPI-----------------------------LDKR    222
S.cerevisiae      VVLCTVGRGKVLLTGPHPEFNVRFMRK-----------------STDKHFLETVVENLKA    241
C.albicans        VVYRKVGKGGVILSGTHPEFAPHLLHP----------------RDEDGAGYFIVVDTLRA    240
Z.tritici         VVYCKVGEGAAVLTGPHPEFCGINLNR----------------DEPSNPDYGHIVDALTA    250
A.fumigatus       VVYCKVGEGAAILTGPHPEYVLSENSRGKLTDATNRFAAANLDKKAGGEEYAKVVDALAA    293
B.cinerea         VVYCKVGEGGAILTGPHPEFAA-----------------VNLDPTIKIPGYSDLVKDLAA    248
C.elegans         LFLYMQNNGS---------LHASALF-SDA----------------------TTQQLIAP    271
D.melanogaster    LAKSLQSGGKAVFSQVHLEMNPSEFE-SDE----------------------TKYSILKQ    666
M.Mulatta         HVPFGTHGGEAVLCQVHLELPPSSDIVQTP----------------------EDFNLLKS    355
H.sapiens         HVPFGTRGGEAVLCQVHLELPPSSNIVQTP----------------------EDFNLLKS    355
M.musculus        HVPFGTLGGEAVLCQVHLELPPGASLVQTA----------------------DDFNVLKS    351
R.norvegicus      HVPFGTHGGEAILCQVHLELPPSAPLVQTT----------------------DDFNVLKS    352
                          *

C.neoformans      SDKARLSWVEELLIKL-------GLTPPQRLTAANRAKSLVDSDKEDPALLLHPTHPSPL    302
S.pombe           DEKTRLELLSYILKLL-------GLKVPKDTSKCGQP------------------TLTDQ    257
S.cerevisiae      QEIMRLKFMRTVLTKT-------GLNCNNDFNYVRAP------------------NLTPL    276
C.albicans        YDHNKKVFMRDCLKKL-------GLRVAESVD-TTIP------------------RVTPM    274
Z.tritici         DDEKRADFMKACLAKL-------GLEVSQETT--PVP------------------SLSHL    283
A.fumigatus       DDKARTDFLKACLSKL-------GLQVTQNTT--TVP------------------SLSSL    326
B.cinerea         DDDSRTKFLKGCLSKL-------GLTVSEEAS--AAP------------------SLSRI    281
C.elegans         N----SNLLKDSLR------SVGVNVCDTTMP----P-----------------LTK-GI    299
D.melanogaster    NERTRLEIFADLLGKYLDVQVRGGDGVDQPQP----G-----------------VVYKHA    705
M.Mulatta         SNFRRYEVLREILTTL-------GLSCDMKQV----P-----------------ALT-PL    386
H.sapiens         SNFRRYEVLREILTTL-------GLSCDMKQV----P-----------------ALT-PL    386
M.musculus        SNVRRHEVLKEILTAL-------GLSCDAPQV----P-----------------ALT-PL    382
R.norvegicus      SNTRRLEVLKEILTAL-------GLSCDAPQA----P-----------------ALT-PL    383
                          .   *

C.neoformans      FFLSHPNL-PQLPEAAVNKPELRGKM----KQ-KDGWNVLRDAND---EIRFGTTEATSP    353
S.pombe           YLFPNNVETKR--FIEKA-------L----TN-----KVKNQDEDTLYTFQFSDIS----    295
S.cerevisiae      FMASAPNKRNYLQEMENN-LAHHGMH----ANNVELCSELNAETD---SFQFYRGY----    324
C.albicans        YVVSPFKD--KVRDVYS-------IL----TS--KLGKSFEDSND---AFYFADET----    312
Z.tritici         HLSSAKPS--DVADLVAT-WREAGIL----TT-ENNSIYIKGEND---TFHLLQT-----    327
A.fumigatus       HLSSQNPA--DTSRIRSS-LQE--VM----SA-EGDGEFIKDEND---TFRLEKP-----    368
B.cinerea         HLSSVHPP--FVPELLAS-LEE--II----EK-ENGEEIIKGEND---TLHLERSE----    324
C.elegans         L-IAEYDSIIES-IAGLRLGEEIGLQPRILLRKSDVVE----------------------    335
D.melanogaster    YFLGRHEAKFEL-------------LEKLRLRCSGSDNVIATPNL---TMKFCGK-----    744
M.Mulatta         YLLSAAEEIRDP-------------LMQWLGKHVDSEGEIKSSQL---SLRFVSS-----    425
H.sapiens         YLLSAAEEIRDP-------------LMQWLGKHVDSEGEIKSGQL---SLRFVSS-----    425
M.musculus        YLLLAAEETQDP-------------FMQWLGRHTDPEGIIKSSKL---SLQFVSS-----    421
R.norvegicus      YLLLAAEEIQGP-------------FMQWLARHVDPEGVIKSSKL---SLKFVSS-----    422

C.neoformans      ERAASEDGITQWLAEAR-RTQPVFPPSIQD--LSIQSDSTPPPPSPPDLHSLTKTILLPS    410
S.pombe           ------------------SEIPEHQLANLD--ISADLSDSDNEIV--------K-IWY--    324
S.cerevisiae      ---------R------------------------ASYDAASSSLLHKEPDEVPKTVIFPG    351
C.albicans        ------------------Q-------------ETSEYVGSEEDPV--KYI-----NFL--    332
Z.tritici         ---------DGWSMASVAQAVAEISP-----KTNATDDTSRDRIL--DYNLVTKTLVP--    369
A.fumigatus       ---------GTWNMSSLEESLPR------------SEQGASEGIV--DYNAIIKPLVI--    403
B.cinerea         ---------SSWSVQSLVKSLSDAVVSTAEEKHTIESAKADDGII--DYNAIPKRVIF--    371
C.elegans         ----------------------------------------ELGLPEATEKLLPIEVVN--    353
D.melanogaster    ----------------------------------------DDKPPVANNNVLPIL-----    759
M.Mulatta         ----------------------------------------YVSEVEITPSCIPVV-----    440
H.sapiens         ----------------------------------------YVSEVEITPSCIPVV-----    440
M.musculus        ----------------------------------------YTSEAEITPSSMPVV-----    436
R.norvegicus      ----------------------------------------YTSEAEITPSSIPVV-----    437
```

```
C.neoformans        PS-VEYSSRWTPLFNFSTYWDELDQARKRSGRRSGVMRQGLDGQAERCSLGDCVLYGETV   469
S.pombe             GDEEKICKKAKPSFDLELYAKLIN----------------------GCRFGLPIIVAPVI   362
S.cerevisiae        VDEDIPPFQYTPNFDMKEYFKYLNV---------------------QNTIGSLLLYGEVV   390
C.albicans          TSAGIPDLKMVPYFDIQKYFDNLRMLS-----------------GGDIKFGSILGYSEVI   375
Z.tritici           HPTSHPDAKSTPHFNHAAFFQHLQTHQSRH-------------PGLAGDYGRTLLYAEVL   416
A.fumigatus         HD-ELPPSKATPYFNHHAFYANLEQYQSQM-------------REGTGCFGSSIMYGEVV   449
B.cinerea           HETDWPSTKETPYFNHHAFYSNLRLYQQEK-------------LSEAEEFGNTLLYGEVV   418
C.elegans           ---RDSDAGTSKNFDFNLYFEQIH-----------------------SKIGQVVLLVDVA   387
D.melanogaster      ---I---HSCPDDFSTVDYFDNLK----------------------TEHIGRLVIYAPVV   791
M.Mulatta           ---TNMEAFSSENFNLEIYRQNLQ----------------------TKQLGKVILFAEVT   475
H.sapiens           ---TNMEAFSSEHFNLEIYRQNLQ----------------------TKQLGKVILFAEVT   475
M.musculus          ---TDPEAFSSEHFSLETYRQNLQ----------------------TTRLGKVILFAEVT   471
R.norvegicus        ---TDPEGFSSEHFNLETYRQNLQ----------------------STRLGRVILFAEVT   472
                                 *.    :   :                           *  :    .

C.neoformans        TSTQTMLDGNPLLLANLPTPLV-FLASFQLSGRGRGSNMWLSPPGCLQFSLLLDLPASL-   527
S.pombe             RSTQTLLDKNYRFLDSTNTGFT-VLGNYQTAGRGRGQNMWVSPYGTLAFSFIINVDAKN-   420
S.cerevisiae        TSTSTILNNNKSLLSSIPESTLLHVGTIQVSGRGRGGNTWINPKGVCASTAVVTMPLQSP   450
C.albicans          TSTNTIMDKNPQWLEHLPNGFT-ITATTQIAGRGRGGNVWVNPRGALATSVLFKIPPSP-   433
Z.tritici           TSTQTILDKNPTWLSHLPIGTT-AVATTQVSGRGRGNNVWVSPPGSLMFTTLLKHPLAL-   474
A.fumigatus         TSTNTILEKNPKLLRKLPHGFT-ATATTQVAGRGRGSNVWVSPAGALIFSTVVRHPVEK-   507
B.cinerea           TSTNTMLEKNPKLLSRLPMGFT-FTATTQIAGRGRGSNVWVSPLGCLIWSVCMKHPMEL-   476
C.elegans           TTTMDIIESVNAGIP-SLESVV-VIANRQISGRGRGGNEFLCPRGMAMFNFSFSISKKS-   444
D.melanogaster      SSSMHLINNLEL-----IHGLA-VLPVQQTSGVGRRNNQWLSPPGCAMFSLQLHLTMDS-   844
M.Mulatta           PTTMRLLDGLMFQTP-QEMGLI-AIAARQTQGKGRGGNAWLSPVGCALSTLLISIPLRS-   532
H.sapiens           PTTMRLLDGLMFQTP-QEMGLI-VIAARQTEGKGRGGNVWLSPVGCALSTLLISIPLRS-   532
M.musculus          STTMSLLDGLMFEMP-QEMGLI-AIAVRQTQGKGRGPNAWLSPVGCALSTLLVFIPLRS-   528
R.norvegicus        STTMSLLDGLMFEMP-QEMGLI-AIAVRQTQGKGRGPNAWLSPVGCALSTLLVSIPLRS-   529
                     ::  :::                    *  * **  * :: * *    .  .

C.neoformans        ----SSKMVFIQYIMALAVCEAIDEDG------------RLGVRIKWPNDIYAEVEGVGG   571
S.pombe             --FSTTPIALFQYLMALAVVRGIREYAPG--------YENIPAFIKWPNDVYVRVDKGG-   469
S.cerevisiae        VTNRNISVVFVQYLSMLAYCKAILSYAPG--------FSDIPVRIKWPNDLYALSPTYYK   502
C.albicans          --SSSSTVVTLQYLCGLALIESILGYGSNVS-GQGVGYEDMPLRLKWPNDIFIMKPEYFK   490
Z.tritici           --STSAPVVFVQYIAALAIVEGIHSYSNSASTQQSKAHASLPVKLKWPNDIYALSSPSAD   532
A.fumigatus         --IQSAPVVFLQYLAAMAVVRGIKSYDVG--------FENMPVKLKWPNDVYALDPENPD   557
B.cinerea           --GNKAPVVFIQYLAAIAIVEAIHSYDKG--------YDTVPIKLKWPNDIYVQDPSKPG   526
C.elegans           --RIAKHLPILQHIFCVALVEAARNLS---------GYPEFPLHIKWPNDLYCE------   487
D.melanogaster      --ALSSRLPLLQHLVGTAIVNSLRSHE---------EYGVLDISIKWPNDIYAN------   887
M.Mulatta           --QLGQRIPFVQHLMSVAVVEAVRSIP---------KYQDINLRVKWPNDIYYS------   575
H.sapiens           --QLGQRIPFVQHLMSVAVVEAVRSIP---------EYQDINLRVKWPNDIYYS------   575
M.musculus          --QLGQRIPFVQHLMSLAVVEAVRSIP---------GYEDINLRVKWPNDIYYS------   571
R.norvegicus        --QLGQRIPFVQHLMSLAVVEAVRSIP---------EYEDINLRVKWPNDIYYS------   572
                           :  .*::    *  ..                  .   :*****::

C.neoformans        T---------------EVGSGKKGKAKLGGILVNTSFVGGKWRIVV--------------   602
S.pombe             -----------------INFQGKQYMKLSGIIVTSNYRKNVLHLVVGCGINVSNLGPTVS   512
S.cerevisiae        RKNLKLVNTGFEHTKLPLGDIEPAYLKISGLLVNTHFINNKYCLLLGCGINLTSDGPTTS   562
C.albicans          SLDDKS------DISATVDGDDEKFVKVSGALINSQFINKTFYLVWGGGVNVSNPAPTTS   544
Z.tritici           P------------------KVADSWTKIAGILVNSSYASADYTLLVGIGLNALNAQPTTS   574
A.fumigatus         ---------------------KKHYTKICGILINSLFSSNEYIAVVGIGINATNASPTTS   596
B.cinerea           ---------------------KREYVKVGGILVNSSYSSGNYDLVVGIGLNIKNAAPTTS   565
C.elegans           -----------------------RSHKVGGMLLQCSTRDDSFRVSIGCGMNVSNDKPTMC   524
D.melanogaster      -----------------------GNQKIGGLVINTTLQGSQAIVNIGSGINLNNSRPTVC   924
M.Mulatta           -----------------------DLMKIGGVLVNSTLMGETFHILIGCGFNVTNSNPTIC   612
H.sapiens           -----------------------DLMKIGGVLVNSTLMGETFYILIGCGFNVTNSNPTIC   612
M.musculus          -----------------------DLMKIGGVLVNSTLMGETFYILIGCGFNVTNSNPTIC   608
R.norvegicus        -----------------------DLMKIGGVLVNSTLVGETFYILIGCGFNVTNSNPTIC   609
                                          *: *  ::
```

```
C.neoformans        ------------------------------------------------------------    602
S.pombe             LNTLVDEWNKNSD---NPRLEKFSFEKLLASVLNQFDRYHRLLLEEGFS-LILPEYYQYW    568
S.cerevisiae        LQTWIDILNEERQQLHLDLLPAIKAEKLQALYMNNLEVILKQFINYGAA-EILPSYYELW    621
C.albicans          LNLVLEKLNEIRRGKGLSPLPPYEPEILLAKLMFTIDQFYSVFEKSGLQ-PFLPLYYKRW    603
Z.tritici           LAQIF---SS-------AGLPPPQLEALLASILVSFEALYARFCRCGWDDGFQEMYYKHW    624
A.fumigatus         LNALASRFVS-------NKSAPITLEKLLARCLTTFEELYTRFLRTGFDREFETIYYDDW    649
B.cinerea           LNTLL----P-------PHLAPITLEKFLARFLTKFETIYKTFCRNGFDRKLEEVYYKHW    614
C.elegans           LNDMLPKEAE----------TRITKEQLIAETINKFTYYMKDYEDNGPE-TFKKKYHEYW    573
D.melanogaster      INDLIREYNT---RVPNNKLPILKYELLIAMIFNEIERLLGEVQNGDFD-SFYALYYSLW    980
M.Mulatta           INDLVTEYNK---QHKAE-LKPLRADYLIARVVTVLEKLIEEFQDKGPN-SVLPLYYRYW    667
H.sapiens           INDLITEYNK---QHKAE-LKPLRADYLIARVVTVLEKLIKEFQDKGPN-SVLPLYYRYW    667
M.musculus          INDLIEEHNK---QHGAG-LKPLRADCLIARAVTVLEKLIDRFQDQGPD-GVLPLYYKYW    663
R.norvegicus        INDLIEEHNK---QNKAG-LKPLQADCLIARAVTVLEKLIDRFQDQGPD-GVLPLYYKYW    664
```

```
C.neoformans        ------------------------------------------------------------    602
S.pombe             LHSNQTVNLASGG----KAIIQGITSDFGFLLAQLLNEN--NEPTTKVVHLQPDGNSFDL    622
S.cerevisiae        LHSNQIVTLPDHGNT--QAMITGITEDYGLLIAKELVSGSSTQFTGNVYNLQPDGNTFDI    679
C.albicans          FHTNQKVDVDNGSGKQRTCIIKGITPDYGLLIAEDV-------ETKKVLHLQPDGNSFDI    656
Z.tritici           LHEGQVVKLEQEGGL--EVRVKGISSDWGMLVVEEVVK----SGRGRRWELMSDGNSFDF    678
A.fumigatus         LHMHQVVTLEEEGGA--RARIKGITRDYGLLLAEELGWD--DRPTGRVWQLQSDSNSFDF    705
B.cinerea           LHTDQIVTLETEGGA--RAIIRGITTDWGLLRAEELGWE--DRPTGKVWELQSDSNSFDF    670
C.elegans           LHSQQEVLLS---DFNERVTIRGID-DDGYLQVRSK------SNPDKIFSIGDDGNTFDM    623
D.melanogaster      LHSGQSVKICLQKDQEKEAEIVGID-DFGFLEVKLP------T--GTIEIVQPDGNSFDM
      1031
M.Mulatta           VHSGQQVHLGSAD--GPKVSIVGLD-DSGFLQVHQE------G--GEVVTVHPDGNSFDM    716
H.sapiens           VHSGQQVHLGSAE--GPKVSIVGLD-DSGFLQVHQE------G--GEVVTVHPDGNSFDM    716
M.musculus          VHGGQQVRLGSTE--GPQASIVGLD-DSGFLQVHQE------D--GGVVTVHPDGNSFDM    712
R.norvegicus        AHRSAPTRFFLLK--F--------------LEVSTR------V-----------------    685
```

```
C.neoformans        -----------     602
S.pombe             MRNLITRKT--     631
S.cerevisiae        FKSLIAKKVQS     690
C.albicans          FKGLVYKKN--     665
Z.tritici           FKGLVKRKV--     687
A.fumigatus         FRGLVKRKV--     714
B.cinerea           LKGLLKRKV--     679
C.elegans           MKGLIRHKY--     632
D.melanogaster      LKGLIIPKYQ-     1041
M.Mulatta           LRNLILPKRR-     726
H.sapiens           LRNLILPKRR-     726
M.musculus          LRNLIVPKRQ-     722
R.norvegicus        -----------     685
```

Figure S13. Sequence alignment of example eukaryotic class III BPL sequences from fungi and other animals. Sequences include the BPL from *Cryptococcus neoformans* (GenBank OXH65462.1), *Schizosaccharomyces pombe* (UniProt O14353), *Saccharomyces cerevisiae* (UniProt P48445), *Candida albicans*,[10] *Zymoseptoria tritici*,[11] *Aspergillus fumigatus* (GenBank KEY77245.1), *Botrytis cinerea*,[11] *Caenorhabditis elegans* (UniProt G5ECZ9), *Drosophila melanogaster* (UniProt Q9VNC3), *Macaca Mulatta* (Rhesus macaque) (UniProt H9EWC5), *Homo sapiens* (UniProt P50747), *Mus musculus* (UniProt Q920N2), and *Rattus norvegicus* (UniProt D4AA38_RAT). Sequences were aligned using Clustal Omega.[12] *Sc*BPL domain architecture is annotated via shading of the *Sc*BPL sequence (pink: N-terminal domain, orange: linker, green: extended catalytic domain region, blue: catalytic domain, light blue: C-terminal cap) and boxes are used to highlight key features (red: catalytic domain GRGRXG and KWPND motifs, purple: N-terminal domain aminotransferase catalytic residues, blue: regions of decreased deuterium exchange for holo-*Sc*BPL compared to apo-*Sc*BPL, with the highest difference in uptake indicated by the box with the bolder line).

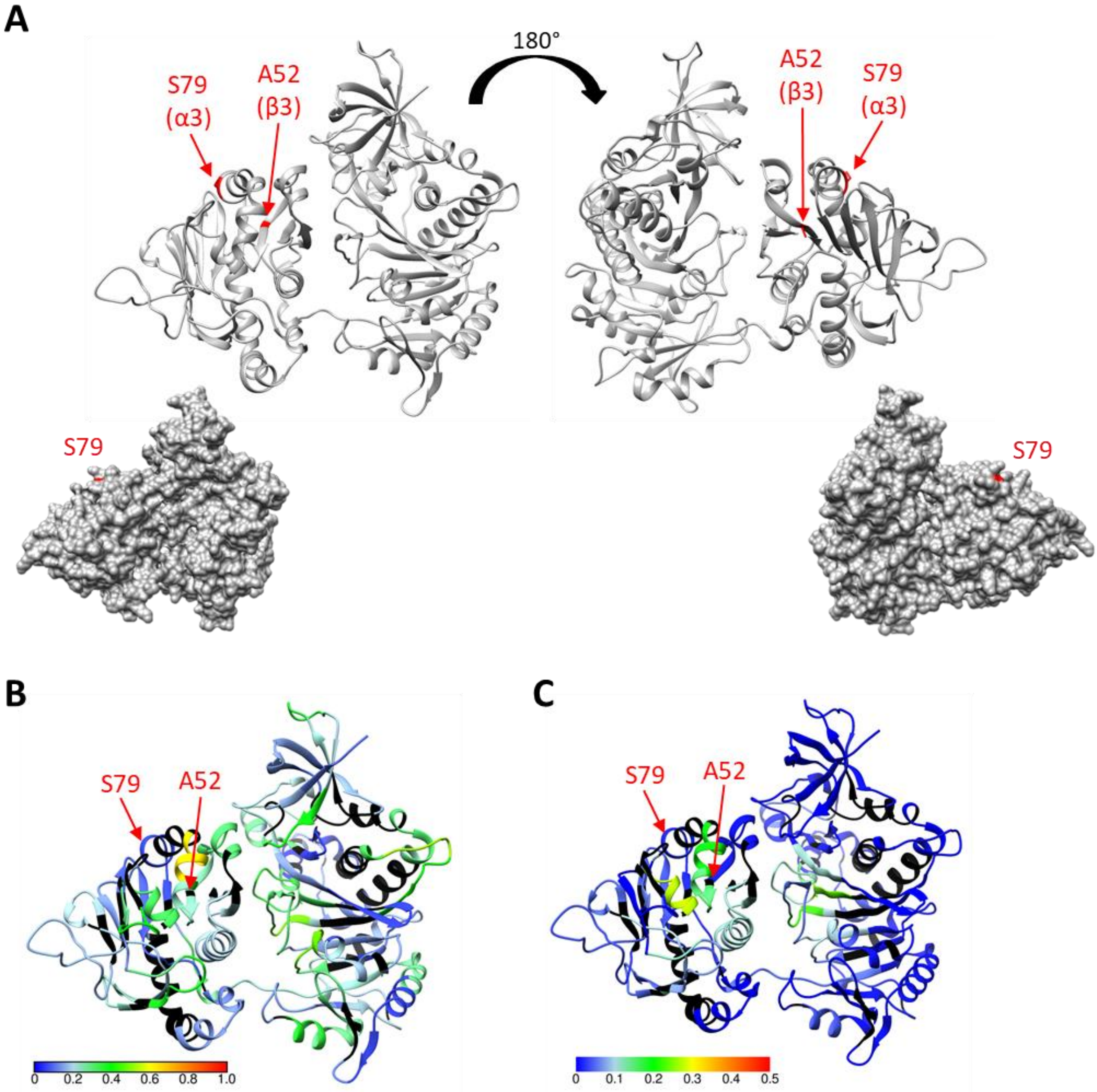

Figure S14. Mapping the *Sc*BPL residues analogous to the human BPL N-terminal domain mutations that cause Multiple Carboxylase Disease (MCD) onto the *Sc*BPL AlphaFold structure (highlighted in red, AF-P48445-F1-v4, UniProt P48445)[1-2]). A) Human BPL N-terminal domain mutations L216R and L237P correspond to residues A52 and S79 respectively, according to protein sequence (Clustal Omega[12]) and AlphaFold model structural alignments. Also shown, in the same structural orientation, are B) the apo-*Sc*BPL HDX results and C) the HDX difference results between apo-*Sc*BPL and holo-*Sc*BPL (as show in Figure 6).

Table S1. Masses of the species observed in the MS spectra of apo- and holo-treated *Sc*BPL.

***Sc*BPL**	**Measured MW (Da)**	**Corresponding ligand-bound state**	**Calculated Mass (Da)**
apo-	77261*	apo, no ligands bound	77244
holo-	77275*	apo, no ligands bound	77244
	77817	holo, reaction intermediate (biotinyl-5′-AMP) bound	77817

*the measured MWs for the apo species are slightly higher than expected (calculated from protein sequence) due to the binding of adducts.

Table S2. Full width half maximum (FWHM) calculated from the IM-MS $^{TW}CCS_{N2}$ distribution data for all charge states (15+ to 18+) for apo-*Sc*BPL and holo-*Sc*BPL.

	$^{TW}CCS_{N2}$ FWHM (nm^2)	
**Charge State**	**Apo-*Sc*BPL**	**Holo-*Sc*BPL**
**18+**	3.27	1.98
**17+**	3.40	2.78
**16+**	4.30	3.49
**15+**	4.15	3.10

Table S3. Structural similarity, measured by RMSD, between the *Sc*BPL C-terminal catalytic domain model produced by AlphaFold (BPL1, UniProt P48445)[1-2]) and crystal structures of Class I and II BPLs from other species.

Species	BPL Class	PDB	Colour	RMSD Å2 between secondary structure cores (and whole length structures)
M. tuberculosis	Class I	4OP0	blue	1.287 for 89 aa (7.751 for 245 aa)
P. horikoshii	Class I	1WPY	purple	1.24 for 108 aa (5.763 for 231 aa)
A. aquifex	Class I	2EAY	green	1.050 for 90 aa (7.702 for 207 aa)
E. coli	Class II	2EWN	red	1.292 for 111 aa (15.115 for 309 aa)
S. aureus	Class II	3RIR	grey	1.240 for 107 aa (20.323 for 304 aa)

An overlay of these structures is presented in Supporting Figure S8.
PDB references for the structures are 4OP0,[3] 1WPY,[4] 2EAY,[5] 2EWN[6] and 3RIR.[7]

Methods

1D ^{1}H NMR for glutamine amidotransferase activity

To determine whether the N-terminal domain had any glutamine amidotransferase activity, either apo- or holo-*Sc*BPL were incubated with glutamine and the 1D ^{1}H NMR spectrum monitored for changes (i.e., decreased glutamine signals and appearance of glutamate resonances). Reactions contained 50 μM DSS, 10% D_2O, 50 mM Tris pH 8.0, 5 mM $MgCl_2$, 1 mM DTT, 20 mM L-glutamine and 1 μM apo-*Sc*BPL. To produce holo-*Sc*BPL, 50 μM biotin and 500 μM ATP were added. The pH of the samples was adjusted to 8.0 prior to NMR analysis. Standard samples of 20 mM L-glutamine and 20 mM L-glutamate in the absence of *Sc*BPL were utilised as controls and as reference chemical shifts.[13]

NMR experiments were carried out on an Agilent Inova 600 MHz spectrometer equipped with a cryo-probe at 298 K. ^{1}H signals were referenced to DSS at 0 ppm. Spectra were processed and analysed using VnmrJ 4.2 (Agilent Technologies). Spectra were collected at the following time points; 1-2 hours, 3 days, 1 week, 2 weeks and 3 weeks, with samples incubated at room temperature to allow reaction catalysis.

In vitro biotinylation activity assays

Assays to measure the biotinylation activity of *Sc*BPL were completed as previously described[11], with the addition of between 0.1 to 47 mM glutamine, glutamic acid or valine (the latter two amino acids were added as controls to compare different amino acid classes). Three technical replicates were employed for every biological replicate. Raw enzyme activity counts were normalised to the *Sc*BPL positive control (no amino acids added) to allow comparison between biological replicates with variable raw activity counts.

References

[1] J. Jumper, R. Evans, A. Pritzel, T. Green, M. Figurnov, O. Ronneberger, K. Tunyasuvunakool, R. Bates, A. Žídek, A. Potapenko, A. Bridgland, C. Meyer, S. A. A. Kohl, A. J. Ballard, A. Cowie, B. Romera-Paredes, S. Nikolov, R. Jain, J. Adler, T. Back, S. Petersen, D. Reiman, E. Clancy, M. Zielinski, M. Steinegger, M. Pacholska, T. Berghammer, S. Bodenstein, D. Silver, O. Vinyals, A. W. Senior, K. Kavukcuoglu, P. Kohli, D. Hassabis "Highly accurate protein structure prediction with AlphaFold", Nature 2021, 596, 583-589.

[2] M. Varadi, S. Anyango, M. Deshpande, S. Nair, C. Natassia, G. Yordanova, D. Yuan, O. Stroe, G. Wood, A. Laydon, A. Žídek, T. Green, K. Tunyasuvunakool, S. Petersen, J. Jumper, E. Clancy, R. Green, A. Vora, M. Lutfi, M. Figurnov, A. Cowie, N. Hobbs, P. Kohli, G. Kleywegt, E. Birney, D. Hassabis, S. Velankar "AlphaFold Protein Structure Database: massively expanding the structural coverage of protein-sequence space with high-accuracy models", Nucleic Acids Res. 2022, 50, D439-D444.

[3] Q. Ma, Y. Akhter, M. Wilmanns, M. T. Ehebauer "Active site conformational changes upon reaction intermediate biotinyl-5'-AMP binding in biotin protein ligase from Mycobacterium tuberculosis", Protein Sci. 2014, 23, 932-939.

[4] B. Bagautdinov, C. Kuroishi, M. Sugahara, N. Kunishima "Crystal structures of biotin protein ligase from Pyrococcus horikoshii OT3 and its complexes: structural basis of biotin activation", J. Mol. Biol. 2005, 353, 322-333.

[5] C. M. Tron, I. W. McNae, M. Nutley, D. J. Clarke, A. Cooper, M. D. Walkinshaw, R. L. Baxter, D. J. Campopiano "Structural and functional studies of the biotin protein ligase from Aquifex aeolicus reveal a critical role for a conserved residue in target specificity", J. Mol. Biol. 2009, 387, 129-146.

[6] Z. A. Wood, L. H. Weaver, P. H. Brown, D. Beckett, B. W. Matthews "Co-repressor induced order and biotin repressor dimerization: a case for divergent followed by convergent evolution", J. Mol. Biol. 2006, 357, 509-523.

[7] N. R. Pendini, M. Y. Yap, D. A. Traore, S. W. Polyak, N. P. Cowieson, A. Abell, G. W. Booker, J. C. Wallace, J. A. Wilce, M. C. Wilce "Structural characterization of Staphylococcus aureus biotin protein ligase and interaction partners: an antibiotic target", Protein Sci. 2013, 22, 762-773.

[8] F. Zein, Y. Zhang, Y. N. Kang, K. Burns, T. P. Begley, S. E. Ealick "Structural insights into the mechanism of the PLP synthase holoenzyme from Thermotoga maritima", Biochemistry 2006, 45, 14609-14620.

[9] L. A. Kelley, S. Mezulis, C. M. Yates, M. N. Wass, M. J. Sternberg "The Phyre2 web portal for protein modeling, prediction and analysis", Nat. Protoc. 2015, 10, 845-858.

[10] N. R. Pendini, L. M. Bailey, G. W. Booker, M. C. Wilce, J. C. Wallace, S. W. Polyak "Biotin protein ligase from Candida albicans: expression, purification and development of a novel assay", Arch. Biochem. Biophys. 2008, 479, 163-169.

[11] L. M. Sternicki, S. Nguyen, K. J. Pacholarz, P. Barran, N. R. Pendini, G. W. Booker, Y. Huet, R. Baltz, K. L. Wegener, T. L. Pukala, S. W. Polyak "Biochemical characterisation of class III biotin protein ligases from Botrytis cinerea and Zymoseptoria tritici", Arch. Biochem. Biophys. 2020, 691, 108509.

[12] F. Madeira, N. Madhusoodanan, J. Lee, A. Eusebi, A. Niewielska, A. R. N. Tivey, R. Lopez, S. Butcher "The EMBL-EBI Job Dispatcher sequence analysis tools framework in 2024", Nucleic Acids Res. 2024, 52, W521-W525.

[13] V. Govindaraju, V. J. Basus, G. B. Matson, A. A. Maudsley "Measurement of chemical shifts and coupling constants for glutamate and glutamine", Magn. Reson. Med. 1998, 39, 1011-1013.
